# Chromosomal rearrangements as a source of new gene formation in *Drosophila yakuba*

**DOI:** 10.1371/journal.pgen.1008314

**Published:** 2019-09-23

**Authors:** Nicholas B. Stewart, Rebekah L. Rogers

**Affiliations:** 1 Department of Bioinformatics and Genomics, University of North Carolina at Charlotte, Charlotte, North Carolina, United States of America; 2 Department of Biological Sciences, Ft Hays State University, Ft Hays, Kansas, United States of America; Fred Hutchinson Cancer Research Center, UNITED STATES

## Abstract

The origins of new genes are among the most fundamental questions in evolutionary biology. Our understanding of the ways that new genetic material appears and how that genetic material shapes population variation remains incomplete. *De novo* genes and duplicate genes are a key source of new genetic material on which selection acts. To better understand the origins of these new gene sequences, we explored the ways that structural variation might alter expression patterns and form novel transcripts. We provide evidence that chromosomal rearrangements are a source of novel genetic variation that facilitates the formation of *de novo* exons in *Drosophila*. We identify 51 cases of *de novo* exon formation created by chromosomal rearrangements in 14 strains of *D*. *yakuba*. These new genes inherit transcription start signals and open reading frames when the 5’ end of existing genes are combined with previously untranscribed regions. Such new genes would appear with novel peptide sequences, without the necessity for secondary transitions from non-coding RNA to protein. This mechanism of new peptide formations contrasts with canonical theory of *de novo* gene progression requiring non-coding intermediaries that must acquire new mutations prior to loss via pseudogenization. Hence, these mutations offer a means to *de novo* gene creation and protein sequence formation in a single mutational step, answering a long standing open question concerning new gene formation. We further identify gene expression changes to 134 existing genes, indicating that these mutations can alter gene regulation. Population variability for chromosomal rearrangements is considerable, with 2368 rearrangements observed across 14 inbred lines. More rearrangements were identified on the X chromosome than any of the autosomes, suggesting the X is more susceptible to chromosome alterations. Together, these results suggest that chromosomal rearrangements are a source of variation in populations that is likely to be important to explain genetic and therefore phenotypic diversity.

## Author summary

*De novo* genes and duplicate genes are a key source of new genetic material on which selection acts. To better understand the origins of these new gene sequences, we explored the ways that structural variation might alter expression patterns and form novel transcripts. These new genes can immediately form new proteins, without the need for multiple mutations during new gene formation. We find that chromosomal rearrangements can form new gene sequences when pieces of genes are copied and moved to new locations. These rearrangements are often found on the X chromosome suggesting rapid shuffling of sex chromosomes in *Drosophila*.

## Introduction

Understanding the origins of new genes is essential for a complete description of evolutionary processes. Mutation generates the raw genetic material that can contribute to phenotypic diversity in natural populations. Without this new genetic material, selection cannot produce change. When new genes are required for adaptation to new and changing environments, where do they come from? How do they arise? Proposed sources of new genetic material include duplicate genes [1, 2], chimeric genes [3–5], *de novo* genes [6–9], and domesticated transposable elements [10]. Deep sequencing of genomes has made it trivial to identify single nucleotide polymorphisms (SNPs) in population genetic data [11, 12]. In contrast, structural variants and duplications remain understudied, in part because they are more difficult to identify in sequence data. With improvements in throughput and quality of next generation sequencing, we can begin to explore the full effects of these complex mutations in nature.

Chromosomal rearrangements contribute to genomic divergence across species. While organisms exhibit striking similarity in genome content, genome organization becomes scrambled over time breaking syntenic blocks [13–15]. Gene movement due to chromosomal rearrangements is known to influence gene expression in primates [16]. Such natural variation from genomic neighborhood is similar to positional effects observed in transgenic constructs [17]. Yet, the implications of these mutations go far beyond quantitative changes in mRNA levels. Mutations that copy and shuffle pieces of DNA can produce new gene sequences. They have the potential to form whole gene duplications, chimeric genes or alternative gene constructs. The full spectrum of new gene creation from these mutations is not fully explored.

Recent work has identified new gene formation through *de novo* exon creation when duplicated segments do not respect gene boundaries [18]. These new genes may ascribe a genetic cause to patterns that mimic *de novo* gene creation. Similar cases of new gene formation were observed when inversions modified gene sequences at breakpoints [19]. However, whole genome studies of rearrangements and new gene formation in natural populations have been lacking. We hypothesize that chromosomal rearrangements may form similar new gene structures when they copy or move pieces of DNA around the genome.

*Drosophila* remain an excellent model system for genomic analysis. Their genomes are compact with little repetitive DNA content, and easily sequenced [20]. Among the *Drosophila*, *D*. *yakuba* houses an unusually large number of chromosomal rearrangements based on reference strain comparisons [20]. Yet, the complexity of population variation for chromosomal rearrangements that might give rise to the observed divergence remains unseen. Here, we use whole genome population resequencing data with paired-end Illumina reads [21] to identify genome structure changes that are segregating in natural populations of *D*. *yakuba*. Pairing these mutation scans with high throughput gene expression data [18], we identify regulatory changes that are produced via chromosomal rearrangements. Across 14 inbred lines of *D*. *yakuba* we used abnormal paired-end read mapping to identify chromosomal rearrangements between and within chromosomes. These mutations may be caused by ectopic recombination, TE movement, inversion formation, template switching during DNA synthesis, ectopic DNA repair, or retrogene formation. These mutations all have the unifying feature that they copy or move DNA from one location to another. We describe the number, types, and locations of rearrangements in population sequence data. Using RNA sequence data from these lines we identified incidences where rearrangements may be creating *de novo* exons and chimeric constructs that create new exons in genomic regions previously devoid of expression. These results suggest that chromosomal rearrangements are a key source of new gene creation that reshapes genome content and organization in nature.

## Results

### Abundance of chromosomal rearrangements

**Fig 1:**
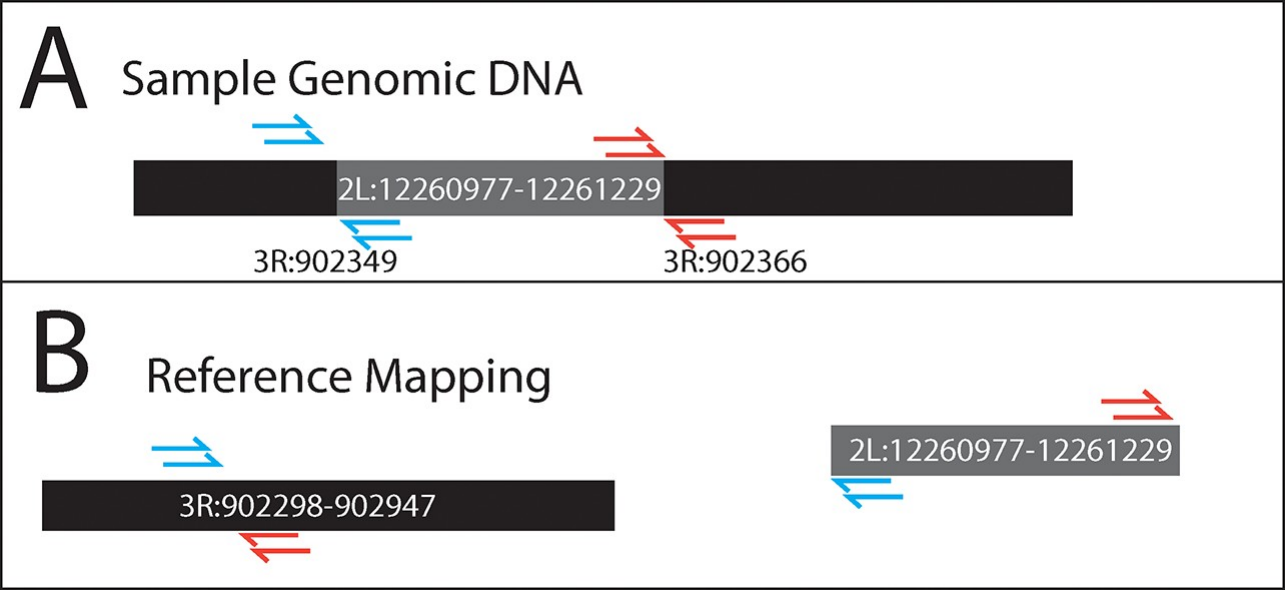
Example of paired end reads mapped abnormally to the reference genome. A) CY17C Chromosome sequenced with paired reads with 325 bp insert size. CY17C has an insertion of sequence from 2L into chromosome arm 3R. B) Each end was then aligned to the reference genome. Left reads that mapped around 3R:902100 had paired right reads mapped to regions near 2L:12261000 (red arrows). Additionally, right reads that mapped around 3R:902600 paired with left reads mapping around 2L:12261150 (blue arrows). This indicates that the region between 2L:12261000–12261150 has been inserted into 3R: 902000–902600 in line CY17C. Each rearrangement needs at least 4 abnormally mapping read pairs to be considered.

We used abnormally mapping Illumina paired-end sequence reads to survey chromosomal rearrangements in a previously sequenced population of *Drosophila yakuba* [21]. We remapped paired-end reads to the reference genome r.1.05 of *Drosophila yakuba* [20] and *Wolbachia* endoparasite sequence NC_002978.6. After removing PCR duplicates, the average depth of coverage of each line varied between 12X to 93X coverage (Table 1). We identified regions that are supported by at least 4 independent read-pairs that map at least 1 Mb away from each other on the same chromosome or on separate chromosomes, similar to methods previously implemented in human genetics [22] (see Fig 1). We identified 2368 total rearrangements among the 14 lines of *Drosophila yakuba*: 1697 rearrangements between chromosomes and 671 within chromosomes. These rearrangements lie within 1kb of 1202 genes.

**Table 1:** Number of chromosomal rearrangements found in the 14 lines. Our ability to identify rearrangements was associated with sequence coverage depth. Number of rearrangements for each line was then predicted at coverage depth of 93.7X coverage using a linear regression model.

Line	Coverage depth	Between chromosomes	Within chromosomes	Total	Between chromosomes (Predicted at 93.7X)	Within chromosomes (Predicted at 93.7X)	Total (Predicted at 93.7X)
*NY73*	27.6	129	57	186	346	129	475
*NY66*	26.5	111	47	158	332	120	452
*NY62*	44.8	177	74	251	337	127	464
*NY48*	34.5	156	59	215	351	124	475
*NY56*	12.1	70	26	96	338	115	453
*NY81*	23.9	119	47	166	348	123	471
*NY85*	61.1	214	82	296	321	118	439
*CY22B*	45.5	148	60	207	306	113	419
*CY21B3*	44.8	155	74	229	315	127	442
*CY20A*	93.7	321	102	423	320	102	422
*CY28A4*	58.3	221	105	326	337	143	480
*CY04B*	64.3	326	129	455	422	161	583
*CY17C*	43.2	171	73	244	337	128	465
*CY08A*	37.5	190	70	260	407	142	549

### Rearrangements as facilitators of new gene formation

Structural variants and duplications can form new exon sequences in regions that were previously untranscribed [18, 22]. These new transcripts appear when gene fragments carrying promoter sequences can drive transcription of new exons with new open reading frames. Formation of such chimeric constructs with *de novo* exons can offer a source of new transcripts within the genome. Like most cases of new gene formation, we expect many of these new variants to be transient rather than stably incorporated into the genome [23–25]. However, as a substrate of genetic novelty, they may occasionally contribute to adaptive changes and phenotypic variation in nature. New genes often appear with expression in the germline [7, 26, 27]. To explore such cases of new open reading frames in the tissues most likely to be affected, we compared rearrangement calls with previously published RNASeq data from testes and accessory glands, male carcasses, ovaries, and female carcasses [18].

**Fig 2:**
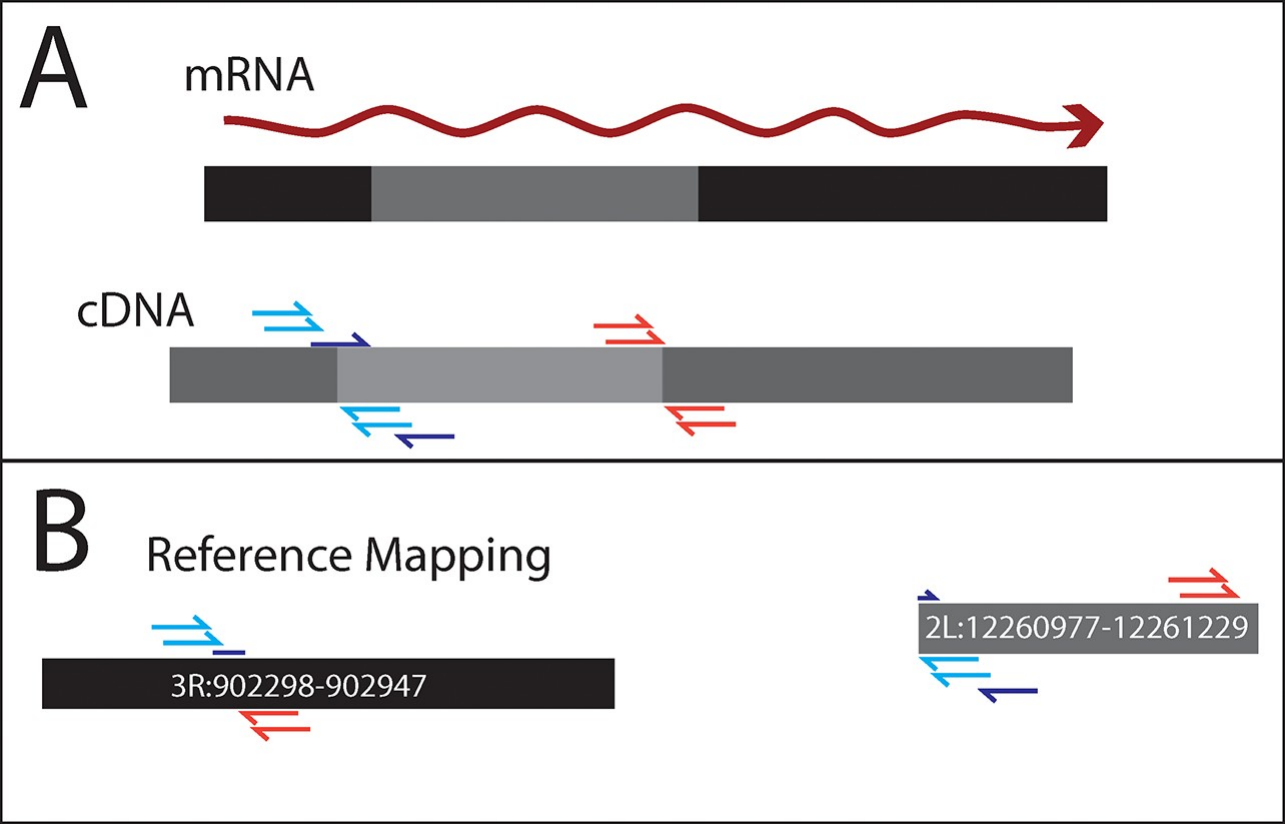
Example of paired end sequence reads mapping from RNASeq data. A) CY17C has an insertion of sequence from 2L into chromosome arm 3R. This insert placed a previously untranscribed region within a previously transcribed gene. Paired end reads were generated from cDNA. B) The paired end reads of this RNA transcript will map to separate chromosomes on the reference sequence, and split read mapping may be seen at the breakpoints. Three total misaligned RNASeq pairs and/or split reads is needed to be considered a formation of a new gene.

**Fig 3:**
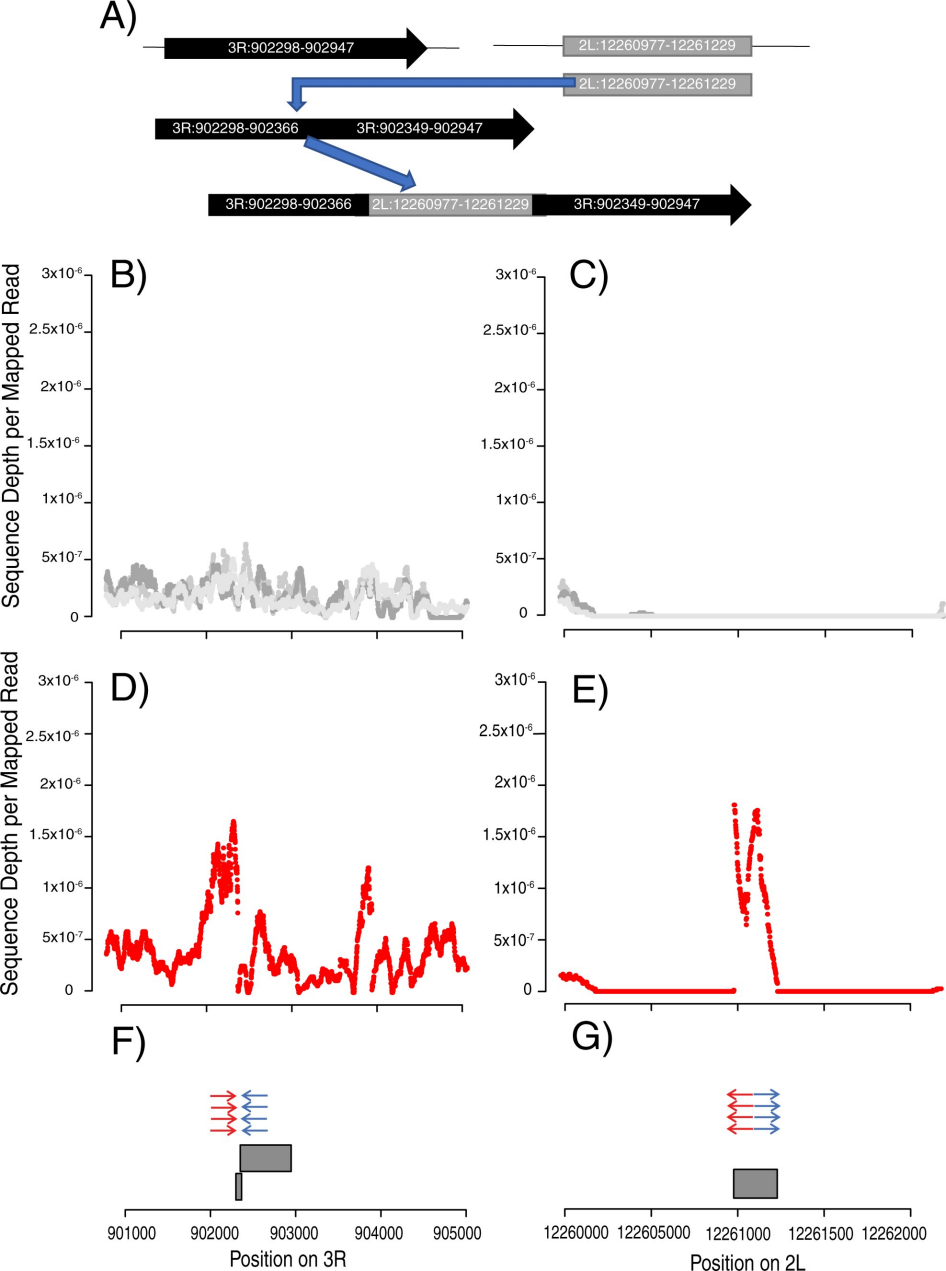
New gene formation through genome rearrangement on chromosome 3R and 2L. Observing sequence depth of the RNA we can infer relative expression and identify newly transcribed regions in lines that have rearrangement calls. Relative RNA Coverage depth was calculated from Tophat RNASeq alignments by dividing the read depth at each base by the total number of reads mapped. Two regions that have 2 genomic rearrangement calls and Tophat fusion calls supporting the formation of a de novo gene. A) Diagram showing the predicted sequence movement based on the Trinity Transcript blast. An insertion of the sequence from 2L:12260976 in-between 902154 and 902563 has moved a segment of previously untranscribed DNA to a region with active transcription on 3R. RNA transcript assembled by Trinity confirms the observed coverage pattern in RNASeq data. The transcript starts near 3R:902000, the middle section mapped between 2L:12260976–12261178 and the final section then maps near 3R:902500. B) and C) The grey coverage lines are RNA sequence coverage from 3 reference RNASeq replicates which do not have this rearrangement. D) and E) RNA sequence coverage of line CY17C which has the rearrangement present. F) and G) CY17C has a two genomic rearrangement calls between 2L:12260976–12261229 matching with 3R:901825–902154 (red arrows) and 3R:902563–902607 (blue arrows). Grey boxes represent the Trinity transcript aligned to the reference genome.

**Fig 4:**
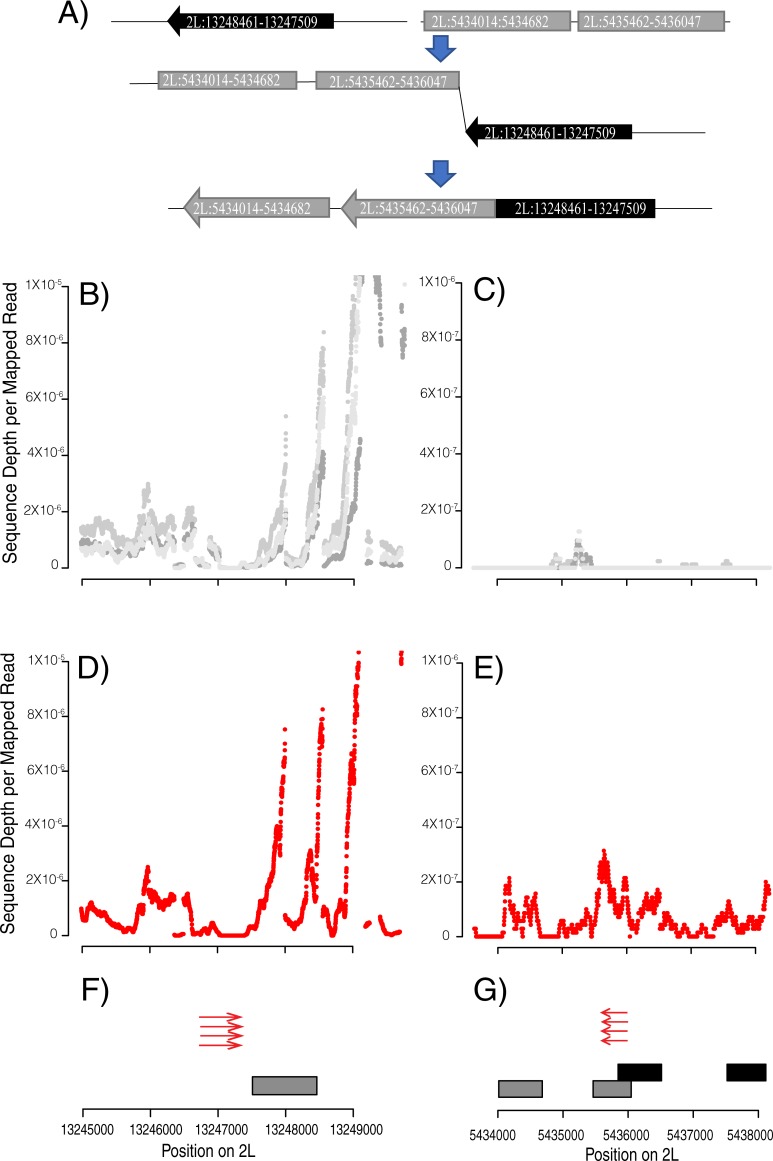
A) Diagram showing the predicted sequence movement based on the Trinity Transcript blast. A rearrangement joining the sequence from 2L:5435633–5436212 902154 and 2L:5435462–5436047 has moved a segment of previously untranscribed DNA to a region with active transcription on 2L. B) and C) The grey coverage lines are RNA sequence coverage from 3 reference RNASeq replicates which do not have this rearrangement. D) and E) CY28A4 has the rearrangement and increased transcription in region 2L:5435462–5436047. F) and G) CY28A4 has a rearrangement calls between 2L:13246986–13247746 matching with 2L:5435633–5436212 (red arrows) and Square boxes in represent the Trinity transcript aligned to the reference genome. The black boxes represent exons of a preexisting gene (1.g484.t1).

To identify new chimeric transcripts formed at rearrangement breakpoints, we used Tophat fusion search [28] to find split reads and abnormally mapping read pairs in RNAseq data (Fig 2). These methods were developed to identify trans-spliced transcripts, but should also identify support for chimeric transcripts produced when genome sequences have been rearranged. We matched structure calls with Tophat fusion [28] calls that are within 1 kb of both sides of rearrangements called in genomic DNA sequences. We used RNASeq read depth to further infer structure of *de novo* transcripts created by chromosome rearrangements (Fig 3) (Fig 4) (S1 Fig) (S2 Fig).

In 14 inbred lines, we isolated 51 putative new genes created by rearrangements. A total of 43 genome structure calls match RNASeq fusion calls that indicate expression in testes. Among such new genes, 32 also show expression in male somatic tissue, while 10 of them are expressed exclusively in the testes. A total of 42 fusion genes were found in male somatic tissue, 33 of which were shared in testes and 9 expressed exclusively in the male somatic tissue. A total of 40 out of 51 transcripts incorporate the start codon of a pre-existing gene (S1 Text). These data suggest that the majority of new genes that form do so by copying or shuffling 5’ promoters and translation signals of genes to drive transcription in new regions. Some 37% (19/51) of the possible new genes identified are singletons (S3 Fig). This pattern is consistent with most of the rearrangements and new gene formation being relatively young and possibly detrimental on average. Additionally, 29% (15/51) of the rearrangements may be ancestral, whereas the reference had a rearrangement that modified a preexisting gene.

**Fig 5:**
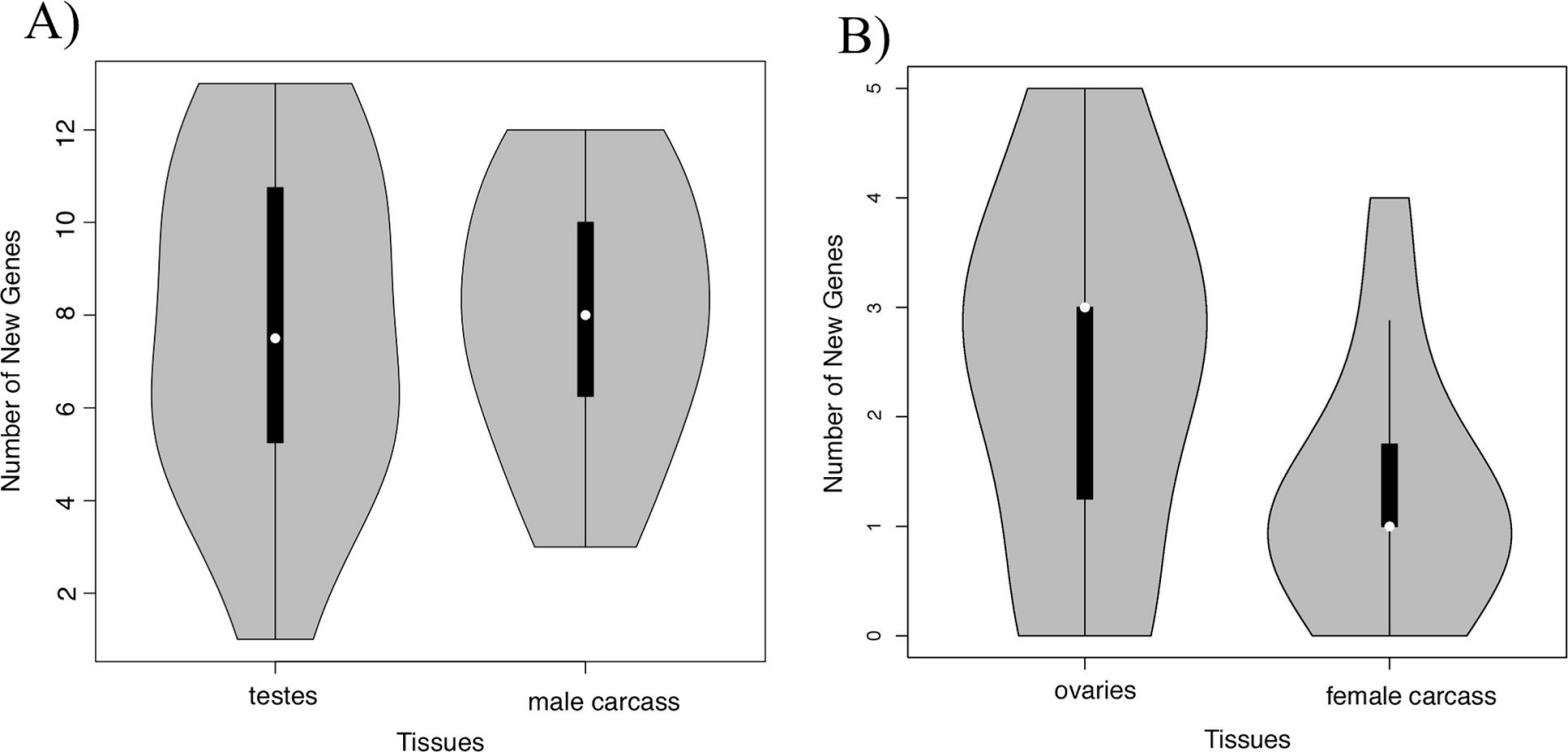
Distribution of new genes per strain identified in testes, male carcass, ovaries, and female carcass based on 14 inbred lines in males (A) and females (B). A total of 51 new genes were identified across all 14 strains in all tissues. We not see a difference in the number of new genes expressed between male gametic and somatic tissue (ANOVA, F(1,13) = 0.04, P>0.8). While there is a significant difference between ovaries and female tissue (ANOVA, F(1,13) = 4.379, P<0.05), the values are low for each line (including being 0 for multiple samples). This suggest that the male comparison is more indicative of the ratio between somatic and gametic tissue.

We observe more new RNA fusion calls at loci with structural rearrangements in testes and male carcasses than ovaries and female carcasses (Fig 5). Testes and male carcass RNA were sequenced using paired-end sequencing while ovaries and female carcass RNA were sequenced using single end sequencing methods. The use of single end data from previously published work reduces the ability to identify fusion transcripts through split read mapping in females (S1 Text). We do not see differences between germline and somatic tissue in males (ANOVA, F(1,13) = 0.04, P>0.8). In females there is a difference between gametic and somatic tissues (ANOVA, F(1,13) = 4.379, P<0.05) though samples sizes are small. In total, we found new transcripts produced by 19 rearrangements expressed in females: 14 expressed in ovaries and 14 expressed female carcass. These data do not allow us to identify every rearrangement in the population, largely due to limits in sequencing coverage. Estimates reported here are conservative, representing the minimum number of instances of new gene formation.

We confirmed cases of new gene formation using reference free transcriptome assembly program Trinity v.2.4.0. These transcriptomes were assembled then aligned using BLASTn to the *D*. *yakuba* reference. A total of 38 *de novo* transcripts (75%) were confirmed by Trinity reference-free transcriptome assembly [29]. However, 5 out of the 13 transcripts that could not be confirmed appear in regions that exhibit multiple rearrangements thus making it difficult to confirm with Trinity. Also, Trinity confirmation rates may be reduced when very small exons fail to align to the reference genome at stringent thresholds (S2 Fig). Thus, 75% represents a minimum confirmation rate.

Of the 38 transcripts that were confirmed with Trinity, start codons were located before the breakpoint in 34 (89%) of the transcripts. This would suggest that most of the putative new genes identified are chimeric. Hence, most rearrangements appear to incorporate the 5’ UTR and start codon of a pre-existing transcript, thereby forming *de novo* exons. These chimeric constructs with *de novo* exons are a source of new transcript formation that can contribute to variation for gene content in natural populations. As with most new gene formation, we expect many of these new genes to be transient [23–25]. However, some small subset may form genetic variation that may be useful for adaptive change.

### Regulatory changes and chromosomal rearrangements

Chromosomal rearrangements can cause expression changes even when exon sequences remain unmodified [16]. To explore such regulatory changes, we used Cuffdiff from the Tophat/Cufflinks gene expression testing suite [30, 31] to identify genes that have significant change of expression compared to the reference strain. We identified 134 genes within 1kb of a rearrangement that had significant expression differences in at least one tissue compared to the reference strain. These include 41 genes in the testes, 51 in male carcass, 50 in ovaries, and 36 in female carcass that show differential expression associated with rearrangements. Most changes in gene expression associated with chromosomal rearrangements produce decreased expression (S2 Table). Such gene expression changes have the potential to induce phenotypic changes in natural populations.

### Population diversity for chromosomal rearrangements

**Fig 6:**
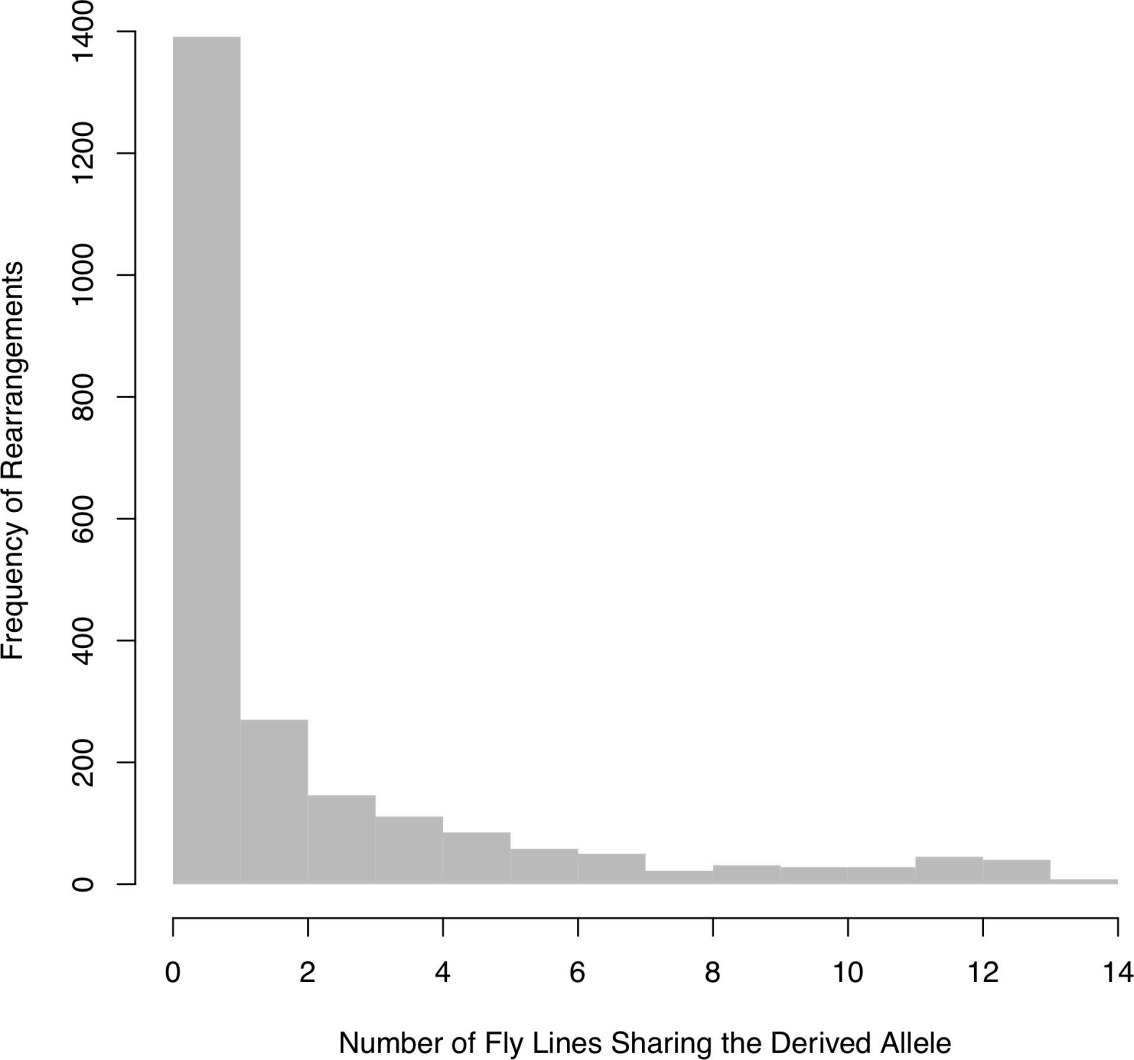
Site frequency spectrum of rearrangements found in the 14 lines. Most of the rearrangements are singletons. However, there is a slight increase in number of rearrangements found in at least 11 of the 14 lines.

The number of rearrangements identified per line varies from 96 to 455 total rearrangements in a single strain (Table 1). Low coverage PacBio long molecule data confirmed 80–97% of mutations per strain suggesting a low false positive rate. Sequencing coverage has a strong effect on false negative rates and confirmation rates (see S1 Text). Mutations were polarized against the ancestral state using a BLASTn against *D*. *erecta*. We identified 112 (4.7%) rearrangements that represent new mutations in the *D*. *yakuba* reference. A total of 54 out of 2368 rearrangements could not be polarized using the existing reference assembly (S1 Text). These are excluded from the site frequency spectrum below. The SFS corrected for false negatives shows that the majority of variants are singletons (Fig 6). This is expected if most of the rearrangements are young and/or have negative fitness.

If rearrangements create novel gene structures or alter gene expression, they may cause phenotypic effects that are subject to natural selection. We wondered whether signatures of selective sweeps might be observed at loci containing rearrangements. Sweep like signals of negative Tajima’s *D* representing highly skewed SFS for the region are not overrepresented among rearrangements (S1 Text). Some rearrangements showed Tajima’s *D* in the bottom 5% of all windows in spite of being singleton variants. These likely represent rearrangements that appeared after the incidence of the sweep. These low frequency variants are not candidates for adaptive changes. However, we observe 10 rearrangements found in at least 75% of lines that are also associated with sweep-like signals (S1 Text). Hence, rearrangements do not appear to be selectively favored as a class, though some individual rearrangements could be adaptive.

### Association with transposable elements

Transposable elements are known to facilitate chromosomal rearrangements in *Drosophila [32]*. They move DNA from one location to another, sometimes creating duplications. TEs can also facilitate ectopic recombination as repetitive sequence mis-pairs during meiosis or mitosis [32]. We compared our rearrangement calls (corrected for false negatives) with TE calls in these lines described previously [21, 33]. We found that 694 rearrangement calls have a TE within 1 kb to one of the sites of the rearrangement and 215 rearrangements have a TE within 1 kb of both sites of the rearrangement. Overall 23.7% (1124/4736) of the rearrangement sites lie within 1 kb of a TE. We found 349 (14.7%) rearrangements have reads that overlap directly with at TE. These rearrangements are confirmed at 86.5–100% in PacBio data, similar the genome wide average. Transposable element content in *Drosophila* is limited compared with other animals. Only 5.5% of the reference genome is composed of TEs [20], though TEs may accumulate in poorly assembled heterochromatic regions. Yet, these selfish genetic elements appear to contribute significantly to polymorphic changes in genome content and organization.

### Genomic distribution of chromosome rearrangement breakpoints

**Fig 7:**
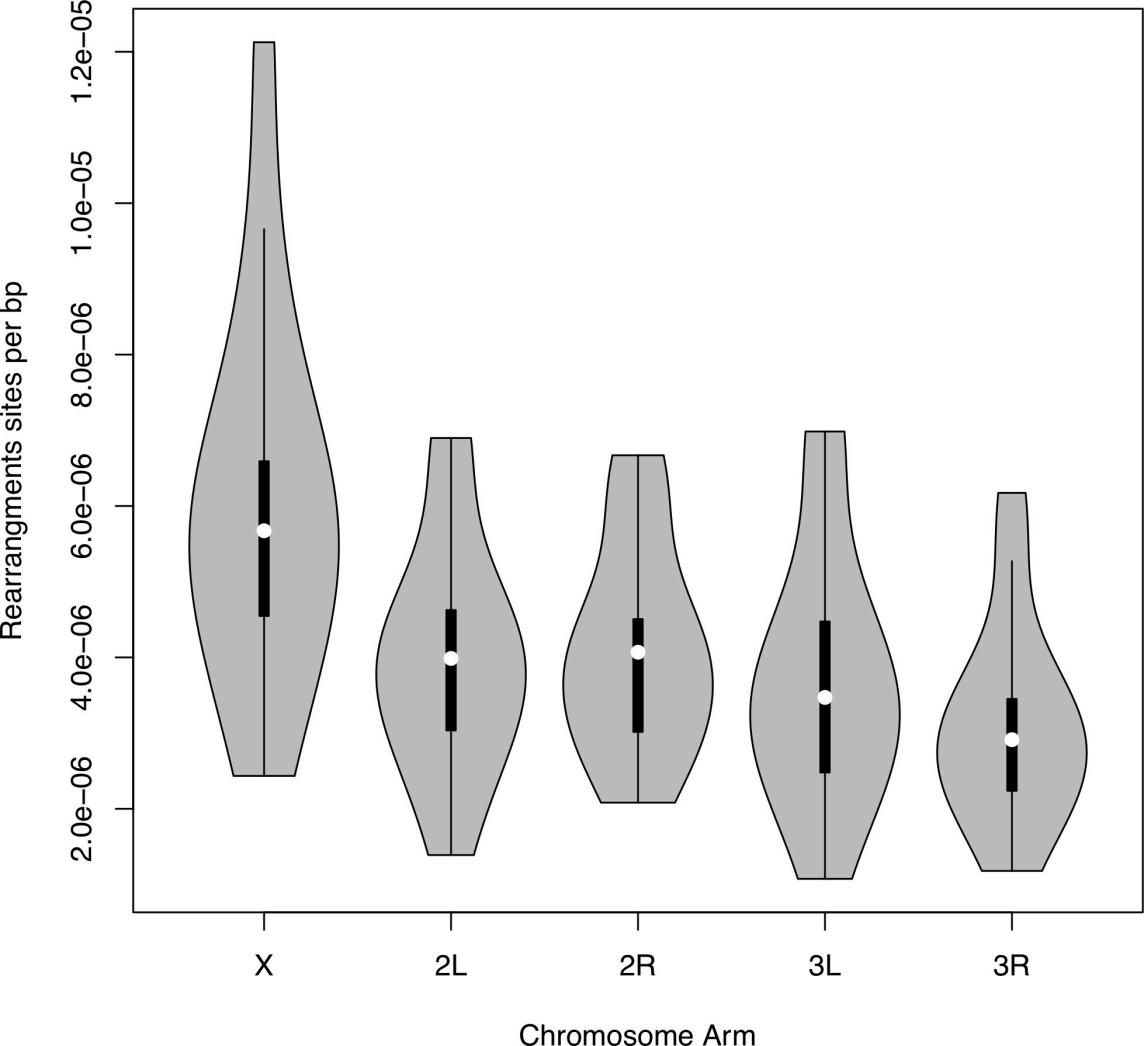
Number of rearrangement breakpoints per base pair on each chromosome arm for inbred lines of *D*. *yakuba*. Total number of rearrangement sites on each chomosome varied (ANOVA, F(4,52) = 43.42, P<10^−15^). This is mostly do to the the fact that the X chromosome has significantly more rearrangement breakpoints than the autosomes (Tukey HSD for each comparison involving the X, *P*<10^−6^). Chromosome 3R had significantly fewer rearrangements than the X, 2L and 2R (Tukey HSD, *P* < 0.05).

Previous work has noted that the X chromosome is a source of newly transposed transcripts, and sex chromosomes are prone to rearrangements due to repetitive content. An excess of genome structure variants involving the sex chromosomes would leave signals of at least 1 breakpoint lying on the X. We identified the distribution of rearrangement breakpoints within each chromosome arm (Fig 7). We standardized the abundance of rearrangements by the length of chromosome arm. We excluded the 4^th^ chromosome (Muller element F) from our analysis. For rearrangements within a chromosome we excluded abnormally mapping read-pairs less than 1 Mb apart. The chromosome arms have unequal abundance of rearrangement breakpoints per base pair (MANOVA, F(4, 52) = 12.35, *P*<10^−11^) (Fig 7). Within-chromosome rearrangements account for 28% of rearrangements, roughly proportional to the amount of the genome housed in a major chromosome arm. These results suggest that the landing place for rearrangements is not biased towards or away from the same chromosome arm.

The X chromosome has significantly more rearrangement breakpoints per base pair than the 4 major autosomes arms (*P*<10^−6^ for each pairwise comparison; S3 Table). The data reveal that 3R has a reduced number of rearrangement sites compared to the X (*P*<10^−7^; S3 Table), 2L (*P*<0.05; S3 Table), and 2R (*P*<0.002; S3 Table). The excess of rearrangements on the X is consistent with previous findings of an abundance of tandem duplications located on the X in *D*. *yakuba* [21]. The X chromosome has more repetitive regions that are more susceptible to ectopic recombination (34, 35). When we distinguish rearrangements based on whether they move DNA across different chromosome arms or affect distant regions on single chromosome arms, the X is still overrepresented (S1 Text, S6 Fig, S4 and S5 Tables).

**Fig 8:**
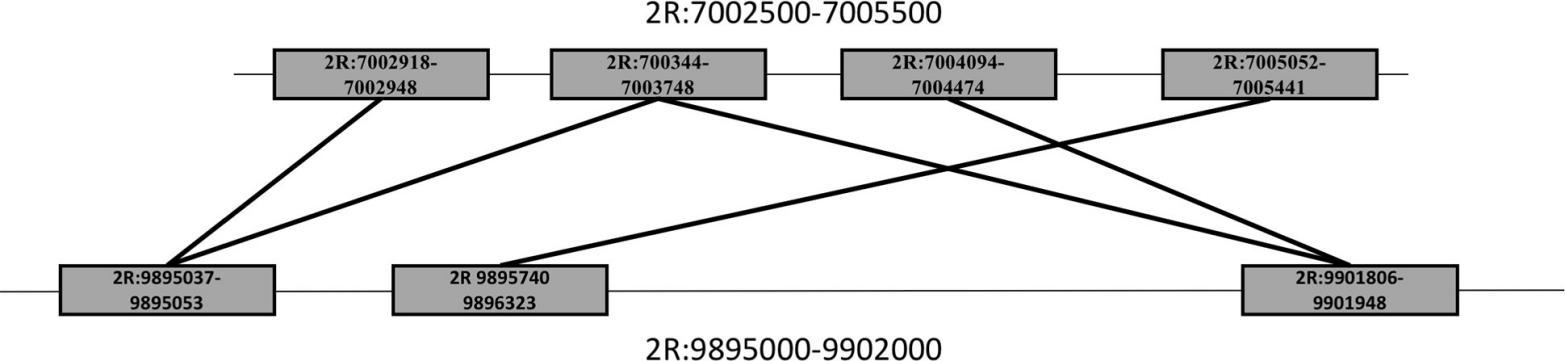
Many rearrangements lie in the same region making it hard to fully elucidate the nature of a particular rearrangement. For instance, in line CY21B3 has 5 rearrangement calls (represented by the connecting lines of the two large sections of chromosome 2R) associated with two regions 2R:7002500–7005500 and 2R:9895000–9902000. 4 separate small regions that are separated by at least 1 sequencing insert size (325 bp) within 2R:7002500–7005500 have reads that pair with 3 separate small regions between 2R:9895000–990200. All the lines always show at least one of these rearrangements but generally each line has 2–3 separate rearrangement calls between regions 2R:7002500–7005500 and 2R:9895000–9902000. The 9.9Mb breakpoint lies close to the known inversion breakpoint on 2R where recombination is suppressed.

We identified 4 ‘hotspots’ of TE movement that had over 30 rearrangement breakpoints in a 5kb span across 14 lines (Fig 8, S7 Fig). One of these hotspots on 2R lies adjacent to a known inversion breakpoint that is expected to suppress recombination. These hotspots contain sequences matching TE families, consistent with TE proliferation (S1 Text). Most rearrangements at hotspots are singleton variants, and each line has fewer than 10 rearrangements. These results suggest recurrent, independent mutations affecting specific regions of the genome.

### Complex variation

Many of our rearrangements are found in clustered pairs, most likely reflecting two breakpoints of an insertion. If the insertion is large enough our methods will separate them into two different rearrangement calls. In other cases where the insertion is small and roughly equal to the read length, our methods make only a single rearrangement call. Some rearrangements appear to be more complex than a simple rearrangement of one sequence transferring to a new location, a challenge for paired-end read mapping. Among the data, one example stands out as an unusually labile region. Chromosome 2R houses a 2.5kb region (2R:7003000–7005000) that has up to 5 rearrangements with a 7kb region 2MB up stream (2R:9895000–9902000) (Fig 8). All lines have at least one rearrangement in this region, and 13/14 of the lines have supporting RNASeq data. This region may have undergone a recent selective sweep (2R:7003000–7005000; Tajima’s *D* = -2.1364) (2R:9895000–9902000; Tajima’s *D* = -1.8177). Due to the multiple rearrangements affecting this single region, it is difficult to localize changes to transcripts and gene expression using Illumina data. This region was identified previously as containing an inversion [34]. The multiple rearrangement calls suggest that the inversion possibly is accompanied by multiple duplication events which is also consistent with targeted analysis of this region [34]. Regions such as this one represents dynamic genome sequence with multiple changes in a short time. Further research of complex regions, especially with emerging long read technology, may allow for a better understanding of how genes are affected by multiple relative recent changes [35]. Such future work may provide an even more complete account for the consequences of chromosomal rearrangements on gene expression and new gene formation.

## Discussion

### Chromosomal rearrangements are a source of standing variation

We used paired-end Illumina sequence reads to identify chromosomal rearrangements in 14 sample strains derived from natural populations of *D*. *yakuba*. We identified genes at these locations they might affect. We identified 2368 rearrangement events within these lines of *D*. *yakuba*, indicating there is a substantial standing variation segregating in populations that may provide genetic material for adaptation.

Standing variation is expected to play a considerable role in evolutionary change and adaptive evolution [36]. This variation provides the genetic diversity for a population to quickly adapt to new niches. We further provide evidence that there is significant variation in the presence and locations of rearrangements affecting the standing variation within populations. Also, it appears that the genetic variation from rearrangements are dynamic complex. Some sites appear to have multiple rearrangement events and copy number changes are observed at some rearrangement breakpoints (S1 Text, S7 Fig). Further sequencing with long read technology would help advance the understanding of complex locations that are subject to multiple structural changes [35].

The conservative nature of our study offers a lower bound on the number of rearrangements that are in the genome. We required that rearrangements be supported with at least 4 abnormally mapping read pairs. There may be other mutations with lesser support that did not meet these thresholds. At least one case of a new gene being formed that did not meet the standards of our conservative approach, despite strong evidence in high coverage RNASeq data (S2 Fig). Hence, the full span of real biological variation is likely to be far richer than the limited portrait described here. Taken together this suggest that new gene formation and regulatory changes are an underestimated source of variation in natural populations.

### Chromosomal rearrangements are a source of new transcripts

Previous theory has struggled to explain the ways that *de novo* genes might derive new open reading frames. The canonical progression of new gene formation suggests that many new genes appear as non-coding RNAs due to spontaneous gain of promoters to facilitate transcription [37]. New transcripts would need to acquire translation signals to become fully formed new protein coding genes [9, 37, 38]. Alternative explanations have suggested that pre-existing ORFs in the genome may be primed for translation even prior to transcription [6, 8]. This mechanism raises the question of how translation signals might be recruited prior to transcription.

Here, we present evidence of *de novo* exons due to chromosomal rearrangements carrying promoters and translation start signals to new locations. New genes that result from such processes offer a clear genetic mechanism to explain new transcription. They also explain how translation signals can be acquired during *de novo* gene creation, changing expression and protein structure of new genes without multiple intermediary steps. The immediate progression to fully fledged coding sequences can explain how new genes form and how they might produce coding sequences without the need for secondary or tertiary mutations. With fewer mutational steps these genes are certain to form new proteins so long as translation start signals are captured. Hence, these mutations can explain the formation of new peptides without the possibility of loss through pseudogenization or deletion during protogene stages. We have identified 51 possible instances of the creation of *de novo* exons created from chromosomal rearrangements. Studies of tandem duplications uncovered over 100 combined new genes and 66 duplicated genes, suggesting that tandem duplications may affect gene novelty more than rearrangements in *D*. *yakuba* [21].

Genetic principles of rearrangements and new gene formation are likely to extend beyond the *Drosophila* model. At least one case of a new exon formation through rearrangement has been documented in humans where gene fragments drive expression on previously untranscribed regions [22]. Hence, understanding of these genetic changes in model organisms is likely to offer important information that can be used for future studies on humans. Chromosomal rearrangements in humans are associated with cancers and infertility [39–42], and associated changes in gene copy number or chimera formation can influence risk of disease or evolutionary potential [43]. Additionally, population diversity for new genetic content is essential to explain phenotypic variation within species in nature. Regulatory effects of gene relocation, new protein formation through chimeric genes, and *de novo* exon formation contribute to genetic changes across organisms. These genetic modifications, including new gene formation serve as a substrate of genetic novelty that is likely to be important for adaptation to new environments. As environments fluctuate, emerging new genetic material may become essential to facilitate phenotypic change. Surveys of standing variation in genome structure and gene content will therefore lead to better understanding of natural variation, adaptation, and disease.

### Chromosomal rearrangements are commonly associated with transposable elements

Chromosomal rearrangements can be the result of multiple mechanisms including ectopic recombination, ectopic DNA repair or gene conversion, template switching during DNA synthesis, and transposable element movement. Transposable elements are a major mechanism of the rearrangements identified. We find 38% (909/2368) rearrangements have at least one TE within 1kb. Less than 5% of the major chromosome arms within *D*. *melanogaster* are transposable elements [44]. Transposable elements have been hypothesized as major players in genetic novelty and catalysts in remodeling gene regulation networks [45]. They often contribute sequence homology that can facilitate ectopic recombination. Yet, only 23.5% (12/51) rearrangements that may have formed a *de novo* exon are associated with TEs. This suggests that another mechanism such as gene conversion or ectopic recombination is responsible for the new genes formed. However, TEs and rearrangements could influence gene expression without changing the transcript. We found 134 genes that have significant differential expression from the reference within 1kb of identified rearrangements. Of these 134 rearrangements that are associated with genes, 74 (55%) are also associated with TEs. This suggests that TEs could be catalysts for the changes in gene expression in the genes that have altered expression in association with rearrangements.

### Genomic distribution

Sex chromosomes are subject to rapid rearrangement due to high repetitive content and selection to relocate gene content to autosomes. Consistent with these patterns, we observe an excess of rearrangements associated with the X chromosome in *D*. *yakuba*. We observe significantly more rearrangement sites on the X chromosome compared to the autosomes. This is consistent with previous findings that show the X chromosome has more structural variants in *Drosophila* [21, 46]. In *D*. *melanogaster* the X chromosome has more repetitive content [47–49], unique gene density [50], and smaller populations size [49, 51]. The X chromosome has lower levels of background selection, and contains an excess of sex specific genes [52, 53] compared to autosomes. Among rearrangements creating new transcripts we do not find an overrepresentation of breakpoints associated with the X chromosome, in contradiction with the “out-of-the-X” hypothesis of new gene formation [7, 26]. Power may be limited to detect these effects with small numbers of new genes. Still, it is clear that X chromosome dynamics are unique, making it a prime resource to investigate the role of rearrangements in genome evolution.

In addition to the excess of mutations on the X chromosome, we identify 4 ‘hotspots’ of recurrent, independent mutation. Here, structural variants reshape variation at a single locus, with multiple low frequency variants segregating at the same region. The fact that single regions are mutated independently with unique breakpoints suggests either hypermutability or dynamics of selection on independent mutations similar to proposed ‘soft sweeps’. A similar set of ‘hotspots’ has previously been noted for TE insertions at the locus of *klarsicht* in *Drosophila* [54] and in the evolution of pesticide resistance [55–57]. Whether this locus represents a region subject to strong selection or is rather exceptionally labile remains to be determined. We observe mutations that rearrange sequences within chromosome arms rather than across independent chromosomes are proportional to the amount of DNA housed within the same chromosome arm. These results contrast with gene conversion data in mammals, showing that within-chromosome rearrangements are favored over cross-chromosome recombination during gene conversion [58].

## Methods

### Fly lines and genome sequencing

We used fastq sequences from previously published genomes (PRJNA215876, also available at https://drive.google.com/drive/u/0/folders/0Bxy-54SBqeekakFpeFBib3BXcVE) of 7 isofemale *Drosophila yakuba* lines from Nairobi, Kenya and 7 isofemale lines from Nguti, Cameroon (collected by P. Andolfatto 2002) [21]. The reference strain is UCSD stock center 14021–0261.01, and the genome sequence is previously described in *Drosophila* Twelve Genomes Consortium (2007). Genome sequencing for the 14 isofemale lines are previously described in ref. 13. Briefly, the wild-caught strains and the *D*. *yakuba* reference stock were sequenced with three lanes of paired-end sequencing at the UC Irvine Genomics High Throughput Facility (http://dmaf.biochem.uci.edu).

### Sequence alignment and the identification of chromosomal rearrangements

We mapped paired-end genomic reads to the reference genome of *D*. *yakuba* r1.5 [20] and the *Wolbachia* endoparasite sequence (NC_002978.6) using bwa v/0.7.12 [59] using permissive parameters to allow mapping in the face of high heterozygosity in *Drosophila* (bwa aln -l 16500 -n 0.01 -o 2). The resulting paired-ends were resolved using “sampe” module of bwa to produce bam files. Each bam file was then sorted using samtools sort v/1.6 [59]. These Illumina paired-end sequences were made with PCR amplified libraries [21]. PCR duplicates can give false confidence in rearrangements through amplification of ligation products that do not represent independent DNA molecules. We used samtools rmdup to remove PCR duplicates. To identify genome structure changes, we used paired-end reads that were at least 1Mb away from each other or located on separate chromosomes (Fig 1). These abnormally mapped paired-end reads indicate possible rearrangements within or between chromosomes. Between 1 Mb and 100kb there may be some rearrangements but there are also inversions, moderately sized duplications (some with secondary deletions). A 1Mb threshold may exclude some variation, but allows greater clarity with respect to mutations and mechanisms that might generate mutations. We selected this stringent cut off to reduce the possibility of inversions being identified rather than translocations. To be considered as a possible rearrangement, a minimum of 4 independent reads must show the same paired-end read pattern (Fig 1). To be clustered together, sets of paired-end reads must be mapped within a distance smaller than the insert size of the library (325 bp) to each other on both rearrangement points. Only rearrangements involving major chromosome arms were considered. All heterochromatic or unplaced chromosomes were excluded.

Sequencing coverage is a major factor in false negative rates [21] (S8 Fig). When 4 supporting read-pairs are required to call mutations, we observe a strong correlation between depth and number of rearrangements (*R*^*2*^ *=* 0.8223, *P*<4.8x10^-6^) (S4 Fig). When only 3 supporting read-pairs are required to call mutations, there is no correlation between depth and rearrangement calls (*R*^*2*^ = 0.009632, *P*>0.3) (S9 Fig). Four lines showed an unexpectedly large number of rearrangements when only 3 supporting read-pairs are used (S9 Fig). These sequence data were collected in early Illumina preparations before kit-based sequencing prep was available. Ligation of multiple inserts with high DNA concentration is likely to have produced this pattern. When 4 supporting read-pairs are required, the number of mutation calls fit into expected relative numbers between the lines negating the effects of errant insert ligation.

### Estimating false negatives and false positives

The number of structure calls is strongly correlated with depth of coverage of each line (S4 Fig). We estimated the number of reads of each line that would be expected at 93.7X coverage using a linear regression model between number read calls and depth of sequencing. In low coverage data, paired-end read may underestimate rearrangement numbers by as much as 50%. All flies sequenced were female. Hence, there should not be significant biases against identification of rearrangements involving the X chromosome compared to the autosomes. The lack of coverage in highly repetitive heterochromatic regions will limit ability to identify rearrangements at those loci. However, our goal was to find rearrangements that change gene structure or expression, while heterochromatic regions are generally less gene dense. Requiring 4 supporting read-pairs may lead to many false negatives in low coverage data. To identify specific cases of false negatives, we surveyed each confirmed rearrangement in each line that did not have a positive call that rearrangement. If these other sample lines had 1–3 reads supporting a rearrangement we considered it a false negative in that sample strain. This is expected to identify many of the false negatives, but there may be false negatives that may not have had a single abnormally paired read supporting it which we failed to identify.

False positive rates were determined by using previously published long read PacBio sequences [21]. PacBio sequencing was done for 4 lines NY73, NY66, CY17C, and CY21B3. This sequencing experiment was done in the early stages of long read sequencing and thus coverage depth for each line is between 5X and 10X. We matched PacBio sequence reads to the *D*. *yakuba* reference using a BLASTn with the repetitive DNA filter turned off and an *E*-value cutoff of 10^−10^. If a single molecule read matched in a BLASTn within 2kb of both sides of the genomic rearrangement call it was considered confirmed. The number of rearrangements that were not confirmed divided the number of the total rearrangements for that line provides us with an estimate of the false positive rate.

### Polarization of the ancestral state

All the rearrangements identified are polymorphic in populations and are expected to be relatively new changes. However, each rearrangement was determined relative to the reference strain. Therefore, it is possible that the rearrangement identified could represent a new rearrangement or the ancestral state that has been rearranged in the reference strain. To polarize the rearrangements, we acquired sequences 1kb upstream and 1kb downstream of each rearrangement site. These sequences were then matched to the *D*. *erecta* reference genome using a blastn [20].

If the two sides of a rearrangement aligned within 2kb of each other on the same chromosome in *D*. *erecta*, it was determined that the rearrangement call is the ancestral allele and the reference has the derived allele. Rearrangements that are shared across species will accumulate nucleotide differences. Therefore, hits must have a minimum of 85% nucleotide identity and must span at least a segment of rearrangement call breakpoints as defined by abnormally mapping Illumina reads. Rearrangements are commonly associated with transposable elements and repetitive element, so if the two sides of a rearrangement map close to each other in more than 10 locations the ancestral state could not be determined.

### Gene expression changes

We used previously published RNA sequences (13, 14) to identify gene expression changes and new gene formation associated with genome structure changes. Briefly RNASeq samples were prepared from virgin flies collected within 2 hrs. of eclosion, then aged 2–5 days post eclosion before dissection. Available data includes ovaries and headless carcass for adult females, and testes plus accessory glands (abbreviated hereafter as testes) and headless carcass for adult males. Sequence data are available in the NCBI SRA under PRJNA269314 and PRJNA196536.

We aligned RNASeq fastq data to the *D*. *yakuba* reference genome using Tophat v.2.1.0 and Bowtie2 v.2.2.9 [60]. We utilized Tophat-fusion search algorithm [61] to identify transcripts that represent fusion gene products either between chromosomes or rearrangements within chromosomes. To confirm fusion events, RNASeq fastq data were assembled reference-free into a transcriptome using Trinity v.2.4.0 [29]. Each transcriptome was then matched to the *D*. *yakuba* reference using a BLASTn with the repetitive DNA filter turned off and an *E*-value cutoff of 10^−10^. All genomic mutations are identified as differences between sample and reference strains. Hence, the RNAseq coverage in the reference serves as a ‘control’ to help identify new genes formed at rearrangement breakpoints.

### Identifying fusion transcripts and gene expression changes

Genomic rearrangement calls were matched to fusion calls from Tophat fusion [61] for testes, ovaries, male carcass, and female carcass. If the two sides of a supported rearrangement were within 1kb of the three Tophat fusion reads or read-pairs (Fig 2), the rearrangement was considered candidate *de novo* exons. Genes annotations in *D*. *yakuba* r1.5 within 1 kb of each location of the RNA supported genomic rearrangement calls were identified. Rearrangements where one side is located near a gene and the other side is not, were of particular interest for the creation of *de novo* exons.

Gene expression at each rearrangement was quantified using coverage depth divided by total mapped reads, analogous to FPKM correction. Each of the four tissues described above (testes, male carcass, ovaries, female carcass) were screened for sequence expression differences associated with the rearrangements. Regions that have unique expression patterns associated with rearrangement calls are considered new transcripts. When a rearrangement brings together a gene and a noncoding locus and there is new transcription in the noncoding region is indicative of new genes.

These new genes were further confirmed using the reference free transcript assembler, Trinity. Each transcript was compared to the *D*. *yakuba* references using BLASTn with the repetitive DNA filter turned off and an *E*-value cutoff of 10^−10^. Transcripts that matched to both ends of the rearrangement was considered confirmation.

We used previously published data [18] from the Cuffdiff program of the Cufflinks differential expression program [30] to search for regulatory changes in genes near chromosomal rearrangements. These data rely on previously published gene and transcript annotations from the same RNASeq data [62]. We compared gene expression of each gene versus the reference strain. Genes that were within 1kb of a chromosome rearrangement call and had significant change from the reference strain were identified.

### Gene ontology

Gene ontology was analyzed using DAVID GO analysis software (http://david.abcc.ncifcrf.gov) [63, 64]. We surveyed for overrepresentation of genes within differing functional pathways. Functional groups with an enrichment score greater than 2 were reported. Functional genetic data for *D*. *yakuba* remains sparse. To determine functional categories represented, we identified *D*. *melanogaster* orthologs as classified in FlyBase and used these as input for gene ontology analysis.

### Differences between chromosomes

We analyzed differences among chromosomes using an ANOVA and Tukeys HSD tests using random block design using line as the treatment blocks. To tabulate rearrangement sites among the chromosomes, each rearrangement that was within a singular chromosome arm counted as 2 sites on that chromosomal arm while rearrangements between chromosomes counted as 1 site on each of the chromosomes involved. Differences between the chromosome arms involving rearrangements within a chromosome arm and between chromosome rearrangements were identified individually using an ANOVA and Tukeys’ HSD tests using the same random block design.

### Population genetics

Estimates of θ_π_, θ_W_, and Tajima’s *D* in 5kb windows for this of D. *yakuba* (https://github.com/ThorntonLab/DrosophilaPopGenData-Rogers2015) were previously described in ref 46. These estimates excluded sites with missing data, ambiguous sequence, or heterozygous sites. We report population genetic statistics for each window containing rearrangements and new genes in the data presented here.

## Supporting information

S1 Text Supplementary text. (PDF)

S1 Fig RNAseq depth at a rearrangement breakpoint suggesting new gene formation rearrangements on chromosome 2L. (PDF)

S2 Fig RNAseq depth at a rearrangement breakpoint suggesting new gene formation via rearrangement on chromosomes 2L and 3L. (PDF)

S3 Fig Site frequency spectrum of rearrangements associated with fusion transcripts. (PDF)

S4 Fig Association between sequence coverage and number of rearrangements identified. (PDF)

S5 Fig Confirmation rates depend on sequence coverage. (PDF)

S6 Fig Number of rearrangements per bp by chromosome. (PDF)

S7 Fig Distribution of rearrangements by chromosome. (PDF)

S8 Fig False negatives vs sequencing coverage. (PDF)

S9 Fig Methods performance under less stringent read-pair support. (PDF)

S1 Table Number of new genes on each chromosome in each strain. (PDF)

S2 Table Number of genes adjacent to rearrangements. (PDF)

S3 Table Comparison of rearrangements per base pair by chromosome. (PDF)

S4 Table Comparison of rearrangements per base pair for rearrangements within chromosomes. (PDF)

S5 Table Comparison of rearrangements per base pair for rearrangements across chromosomes. (PDF)

S6 Table Total number of rearrangements with coverage increases. (PDF)

S7 Table Rearrangements with parallel read pairs. (PDF)

S8 Table Gene ontology terms for genes associated with rearrangements. (PDF)

S1 Data Supporting data archive. (ZIP)
