# Supplementary material for: Chromosomal rearrangements as a source of new gene formation in Drosophila yakuba
Source: PLoS Genet. 2019 Sep 23;15(9):e1008314. doi: 10.1371/journal.pgen.1008314 (PMC6776367; doi:10.1371/journal.pgen.1008314)
Supplement: S1 Text — (PDF) [file pgen.1008314.s001.pdf]

S1 Text

Supplementary Information

Types of rearrangements
We classified the rearrangement identified as putative translocations. These genetic changes may encompass any mutation that moves DNA from one location to another. They will include ectopic gene conversion, ectopic recombination, TE movement, repair of staggered breaks, template switching during replication, and putatively retrogenes formation. We classified the rearrangements that were associated with transposable elements, duplications, and inversions

Duplications associated with rearrangements

Transposable element proliferation and duplicative rearrangements can increase genome content by creating extra copies of DNA sequences. To determine the propensity of these rearrangements to create duplicates, we calculated the average depth of coverage of each rearrangement plus 100 bp upstream and downstream and 500 bp upstream and downstream of each rearrangement. We classified possible duplications associated with rearrangements by identifying the rearrangement sites that have average sequence coverage depth that is twice the average coverage depth of that line. Duplications are associated with 5-25% of the rearrangement sites within each line (S6 Table). Line NY56 has an elevated number of possible duplications, however this is most likely associated with the low average coverage (12.1X) skewing the results. Overall, we observe that the majority of the rearrangement sites are not associated with duplications (S6 Table).

More rearrangement sites appear to be duplicated in 100 bps flanking the rearrangements than 500 bps regions flanking the rearrangement sites and as the sequence depth decreases moving away from the rearrangement. The observed coverage differences suggest increased coverage more immediate to the rearrangement suggesting more small duplications around the rearrangement sites than large ones. These results are consistent with other studies suggesting *D. yakuba* has many small tandem duplications (21)**.** It may also be consistent with target site duplications at breakpoints as has previously been observed for inversions (34).

Association with inversions
Previously, 29 inversions were identified in *D. yakuba* (32). We identified rearrangement calls from our dataset that were within 500KB of the estimated breakpoint region of each inversion. Overall, we found 5.3% (36/671) of the rearrangements within a singular chromosome matched to previously identified inversions. Additionally, our rearrangement calls only associated with 55% (16/29) of the previously identified inversions. The methods described here were not intended to identify inversions, which often have complex rearrangements and repeats at their breakpoints. Furthermore, it is possible that some inversions are not present among the strains sequenced here.

Polymorphic inversions within *D. yakuba* would appear as possible rearrangements within our study. In the case of an inversion, the misaligned read pairs would mark inversion break with read pairs would be mapped in the same direction. We do see evidence that within each line roughly 40-60% (mean 51%) (S7 Table) of the rearrangements on the same chromosome do have read pairs that are mapped in the same direction. However, this could be an indication of a translocation when the insert was inserted in reverse rather than a traditional inversion with two breakpoints encompassing all intervening sequence. We note that these numbers are consistent with a 50/50 split of strand orientation for translocations. Rearrangement

between chromosomes cannot be inversions. These show that within each line a similar proportion of 40-55% (mean 47%) of rearrangements having reads in the same direction. If we identify many inversions, then we would expect a higher proportion of the same read pairs in rearrangement within rearrangements than between chromosome structure calls. This combined with that overall roughly 5% of our rearrangements match previously identified inversions suggests that the majority of the rearrangements that have been identified were most likely not inversions. Previous analysis of polymorphism did not note signals consistent with these mutations representing inversions on chromosome arms but did identify atypical SNP patterns in regions matching known inversions on 2R (32, 34).

Previous work on these sequence data estimated that sequencing under 30X coverage would significantly reduce the effectiveness of identifying tandem duplications (21). Our results suggest that this also applies with chromosomal rearrangements. The number of chromosomal rearrangements identified is significantly correlated with sequence depth (R^2=0.8231, P<$4.7x10^{-6}$) (S4 Figure). We used a linear regression to correct the total rearrangements identified in each line based on coverage (Table 1). After corrections for coverage, we would expect between 419 and 583 rearrangements per strain at 94X coverage (Table 1).

Previously published PacBio sequencing was available for four of the lines: NY73, NY66, CY21B3, and CY17C (21). PacBio sequence reads that match using BLASTn within 2kb of both rearrangement sites were considered confirmation of the rearrangement. Between 80%-97% of the rearrangements per strain identified using paired-end Illumina sequence reads could be confirmed in the PacBio long molecule sequencing. Confirmation rates vary according to sequence coverage depth of the PacBio sequencing (R^2=0.9883, P<0.004) (S4 Figure). The confirmation rate of each line is not correlated with the sequence depth of the Illumina sequencing, implying that false positive rate from paired-end read mapping does not depend on coverage. To further analyze false positive, we analyzed rearrangements that are associated with transposable elements at both breakpoints. Transposable elements have the best likelihood to create false positives by misalignments. Overall in these four lines, PacBio reads confirmed at 88% of such rearrangements in each line. The high number of confirmed rearrangements suggests that the number of false positives identified by Illumina sequencing may be under 5% of the total number of rearrangements identified, consistent with previous work (21, 22).

Quality Control for new gene formation

To further evaluate differences between paired end and single end sequencing, we down sampled RNAseq data for males to determine performance using single end reads. Our rearrangement calls using single end reads are reduced from 43 to 21 in testes and from 42 to 16 rearrangements in male carcasses, suggesting much greater power in paired-end read data. New gene formation per line varies from 3 new transcripts (NY48) to 16 (NY62, NY66). No *de novo* transcripts exist that were expressed in ovaries or female somatic tissue that were not also expressed in either testes or male somatic tissue. It is likely that lack of female-specific genes is due to lower power to identify fusion transcripts, though previous work has noted *de novo* genes in *Drosophila* are male-biased (7, 8). We identified putative false negatives as rearrangements in lines with less than 4 support reads that match a rearrangement call in another line. This analysis reveals another 6 constructs, for a maximum of 58 new genes. Additionally, we identified one locus that fails to meet the threshold of 4 independent Illumina sequences, but appears to be a new gene with strong evidence in high coverage RNASeq data (S2 Figure). This new locus shows strong upregulation matching the inferred gene structure from a putative rearrangement supported by 3 read-pairs in genomic data.

Of the 39 transcripts that were confirmed with Trinity, possible start codons were located before the breakpoint in 34 (87%) of the transcripts. Of the 13 rearrangements that were not confirmed with Trinity, 6 of them fell within a previously annotated gene. In all 6 cases the start codon appears to be before the rearrangement breakpoint. Of the other 7 rearrangements 4 appear to have break points within 300 base pairs of previously annotated genes. However, we cannot pinpoint the exact breakpoint of the rearrangements to single bp resolution. This could indicate that regulatory elements have moved but not exons. The final 3 rearrangements, neither break was within 1000 base pairs of a known gene. This could indicate that this rearrangement created a *de novo* gene without the incorporations of existing exons or an incomplete annotation within the *D. yakuba* genome.

Genomic Distribution of Rearrangements

We wondered whether there might be 'hotspots' for rearrangements across the genome. Four hotspots were identified: one on the X, one on 2R, and two on 3R (S7 Figure). One was classified as having over 30 rearrangement breakpoints across 14 lines within a 5kb window (e.g. Fig 8). Interestingly, these rearrangement hotspots were not specific to a particular line. Each hotspot had structure calls associating it with a range of locations and each line has less than 10 rearrangements associated with each region. In aggregate, the population genomic data suggests repeated, independent mutations affecting the same regions in different strains. Most of the rearrangements associated with hotspots are singletons in the data set. The hotspot on 2R close to position 10Mb lies adjacent to a known inversion breakpoint which has been shown to suppress recombination (34).

Multiple rearrangements in the same region suggested action by transposable elements that have independently moved in the lines to unique locations. None of the four rearrangement hotspots were associated with TE calls in a previously reported dataset (33). However a BLAST comparison of sequences to Repbase (65) showed high matching with a TE family including *CMC-Transib*, *Mariner*, and *Jockey* families. Several factors may explain these previously unreported TE calls. Mapping criteria that allow for a greater number of mismatches to capture heterozygosity may allow for additional read mapping than previous strict matches. New releases of bwa may process alignments differently. Also, previous TE calls required breakpoint assembly of the TEs to classify the locus as a transposable element (33), while analysis here does not. The four hotspots all showed similar trends suggesting that highly complex cases of TE movement may have breakpoints that are difficult to assemble in short read data.

Population Genetics of Rearrangements

Paired-end read mapping identifies rearrangements based on differences compared with the reference. It does not identify ancestral states. To determine whether mutations were derived or ancestral, we used BLASTn to compare sequences 1kb downstream and 1kb upstream of each rearrangement site to *D. erecta.* If the two regions matched a single region within 2kb of one another in *D. erecta* then the derived allele is present in the reference while the sample strains contain the ancestral state. We identified 112 rearrangements or 4.7% of the rearrangements that represent new mutations in the *D. yakuba* reference rather than in sample strains. A total of 54

out of 2368 of the rearrangements had pairs that matched over 10 different sites making it too difficult to confidently identify the ancestral and derived allele in the face of repetitive DNA. These were excluded from the site frequency spectrum (SFS).

Of our 2368 rearrangements, we identified 167 (7.1%) have at least one of the rearrangement sites associated with a Tajima's *D* in the bottom 5% (-2.27) of Tajima's *D* measure throughout the major chromosome arms. However only 170/4621 (3.7%) sites have a Tajima's *D* of less than -2.27. We used a bootstrap approach to determine whether sweep-like signatures were overrepresented at these rearrangements. We randomly sampled windows and recorded Tajima's *D* 4621 times along the major chromosome arms with 10,000 replicates. All replicates had at least as many random windows with Tajima's *D* less than -2.27, suggesting rearrangements are underrepresented in regions with signatures of strong, recent selection. This suggest that these rearrangements are most likely to be deleterious or neutral. Our ability to detect selection on our samples might be diminished if hotspots, which vary across lines, bias results to regions with high amounts of segregating variation. It is also possible that these sites could be subject to soft sweeps, which are not easily detected by reduced diversity or site frequency spectra skewed toward rare alleles (66).

While certain rearrangements identified with very low Tajima's *D* are new singleton rearrangements, our analysis could be identifying artifacts of selective sweeps before the appearance of the rearrangement. Derived rearrangements found at high frequencies are may be candidates for selectively favored variation in the population. We identified 125 derived rearrangements found in at least at least 75% (11/14) of the lines. These rearrangements are within 1 kb of 78 genes, 52 of which have orthologs in *D. melanogaster*. These genes have functions in many functional groups including Rho GTPase activity and imaginal disc and wing vein morphogenesis. Of 125 rearrangements 10 (8%) rearrangements had at least one side of the rearrangement has a Tajima's *D* in the lowest 5% (Tajima's $D < -2.27$). However, the overall number of rearrangement sites found in at least 75% of the lines are not overrepresented (11/241, 4.6% have Tajima's $D < -2.27$). Random sampling of 241 windows along all the chromosomes with 10,000 bootstrap replicates reveals that our sample is well in the expected range (1-28) with 30-40% of the trials match or have fewer sites within the bottom 5% of Tajima's *D*.

Gene ontology

To characterize the functional categories that might be affected by these mutations, we explored gene ontology (GO) categories of genes associated with the rearrangements. We used DAVID GO analysis software (63, 64) to investigate the genes being altered by chromosomal rearrangements. We identified 1202 prospective genes within 1kb of a rearrangement call. We identified 733 genes that have a *D. melanogaster* ortholog and are within 1 kb of a rearrangement site. Overrepresented functional categories include alternative splicing, transmembrane proteins, protein phosphorylation, and glycoproteins (S8 Table).

We identified 52 genes within 1 kb of the estimated breakpoints of structure calls that are supported by RNASeq Tophat fusion calls. These loci are candidates for new gene formation. A total of 37 of these genes have orthologs in *D. melanogaster*. These genes however showed no overrepresentation with respect to gene ontology or functions.
