# Supplementary material for: Chromosomal rearrangements as a source of new gene formation in Drosophila yakuba
Source: PLoS Genet. 2019 Sep 23;15(9):e1008314. doi: 10.1371/journal.pgen.1008314 (PMC6776367; doi:10.1371/journal.pgen.1008314)
Supplement: S1 Fig — (PDF) [file pgen.1008314.s002.pdf]

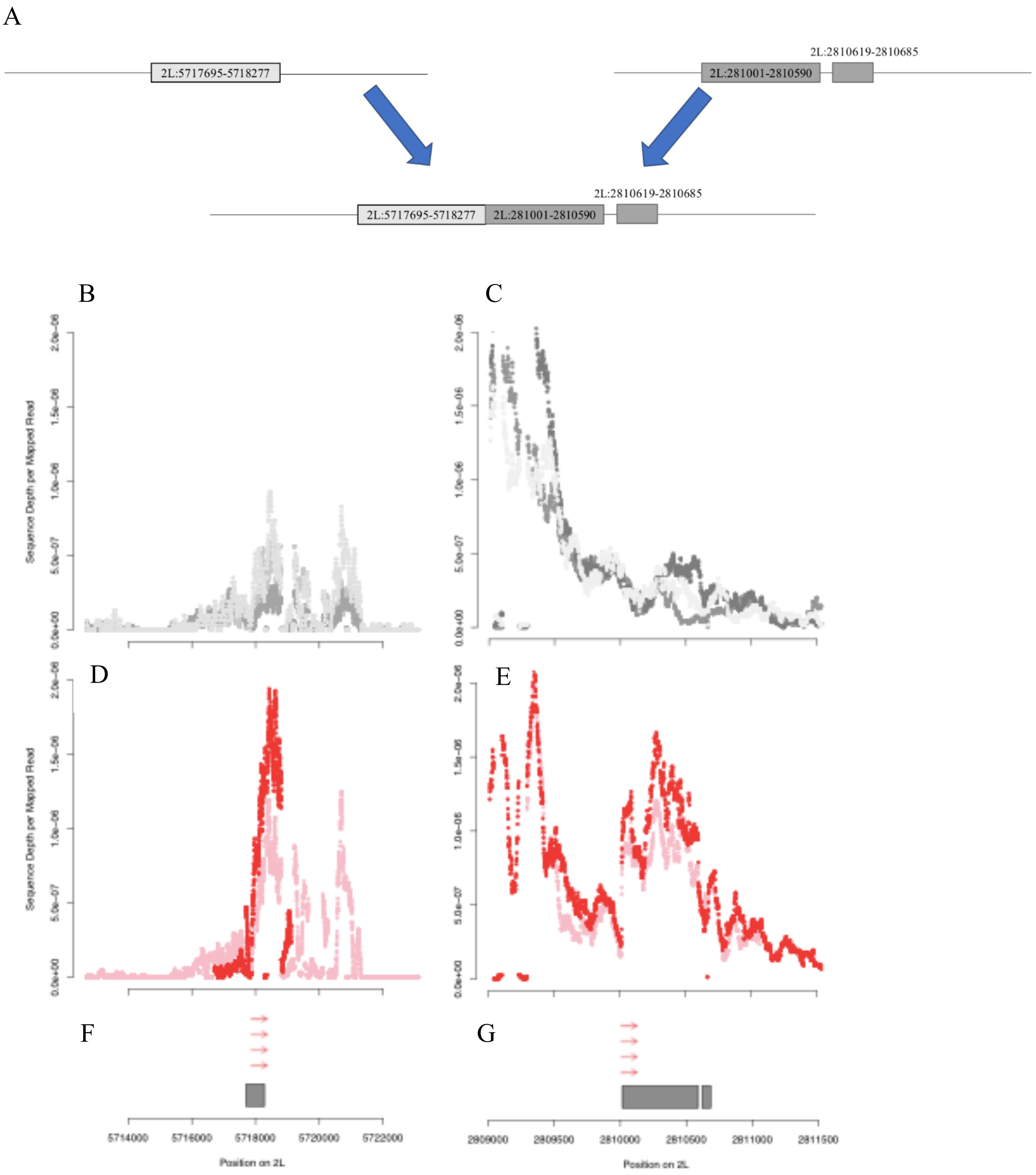

S1 Figure: New gene formation through genome rearrangement on chromosome 3R and 2L. The gene (*GE18374*)/regulatory elements at 2L:5171796-5718110 appears to be altering expression at 2L:2810011-2810533. A) Diagram showing the predicted sequence movement based on the Trinity Transcript blast. Observing sequence depth of the RNA we can infer relative expression and identify newly transcribed regions in lines that have rearrangement calls. B) and C) The grey coverage lines are RNA sequence coverage from Tophat of three replicates of the reference line

male carcass and 3 replicates of reference line testes which do not have this rearrangement. D) and E) CY17C male carcass (red line), testes (pink line) has a rearrangement between 2L:5171796-5718110 and 2L:2810011-2810533. F) and G) Red arrows indicate the genomic rearrangement calls, both reads point in the same direction that suggest the transported section of DNA was inserted inversely. The grey boxes represent the Trinity transcript mapped to the reference genome.
