# Supplementary material for: Chromosomal rearrangements as a source of new gene formation in Drosophila yakuba
Source: PLoS Genet. 2019 Sep 23;15(9):e1008314. doi: 10.1371/journal.pgen.1008314 (PMC6776367; doi:10.1371/journal.pgen.1008314)
Supplement: S2 Fig — (PDF) [file pgen.1008314.s003.pdf]

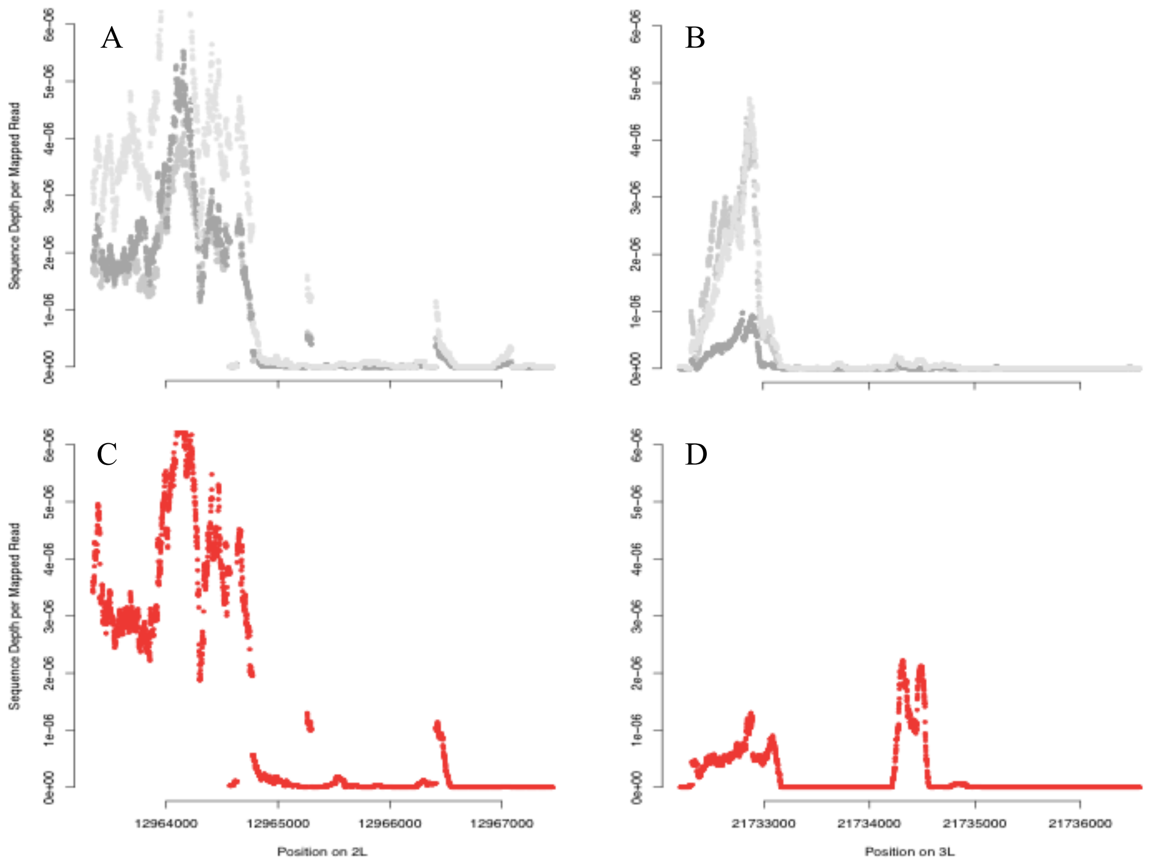

S2 Figure: A) and B) The grey coverage lines are RNA sequence coverage from Tophat of 3 replicates of reference line testes which do not have this rearrangement. C) and D) CY22B testes has a rearrangement between 2L:12965344-12965453 and 3L:21734209-21734559. This rearrangement appears to have created a newly transcribed region 3L:21734209-21734559. This may have been altered by the gene *GE26196* or its surrounding regulatory elements near 2L:12965344-12965453. However, this rearrangement only has 3 supporting Illumina genomic reads. A 4th read pair can be found 25bp over our 325bp coupling limit. This suggest that our conservative approach to the identification of new genes is an underestimate of the overall new genes created by rearrangements.
