# Supplementary material for: Chromosomal rearrangements as a source of new gene formation in Drosophila yakuba
Source: PLoS Genet. 2019 Sep 23;15(9):e1008314. doi: 10.1371/journal.pgen.1008314 (PMC6776367; doi:10.1371/journal.pgen.1008314)
Supplement: S3 Fig — (PDF) [file pgen.1008314.s004.pdf]

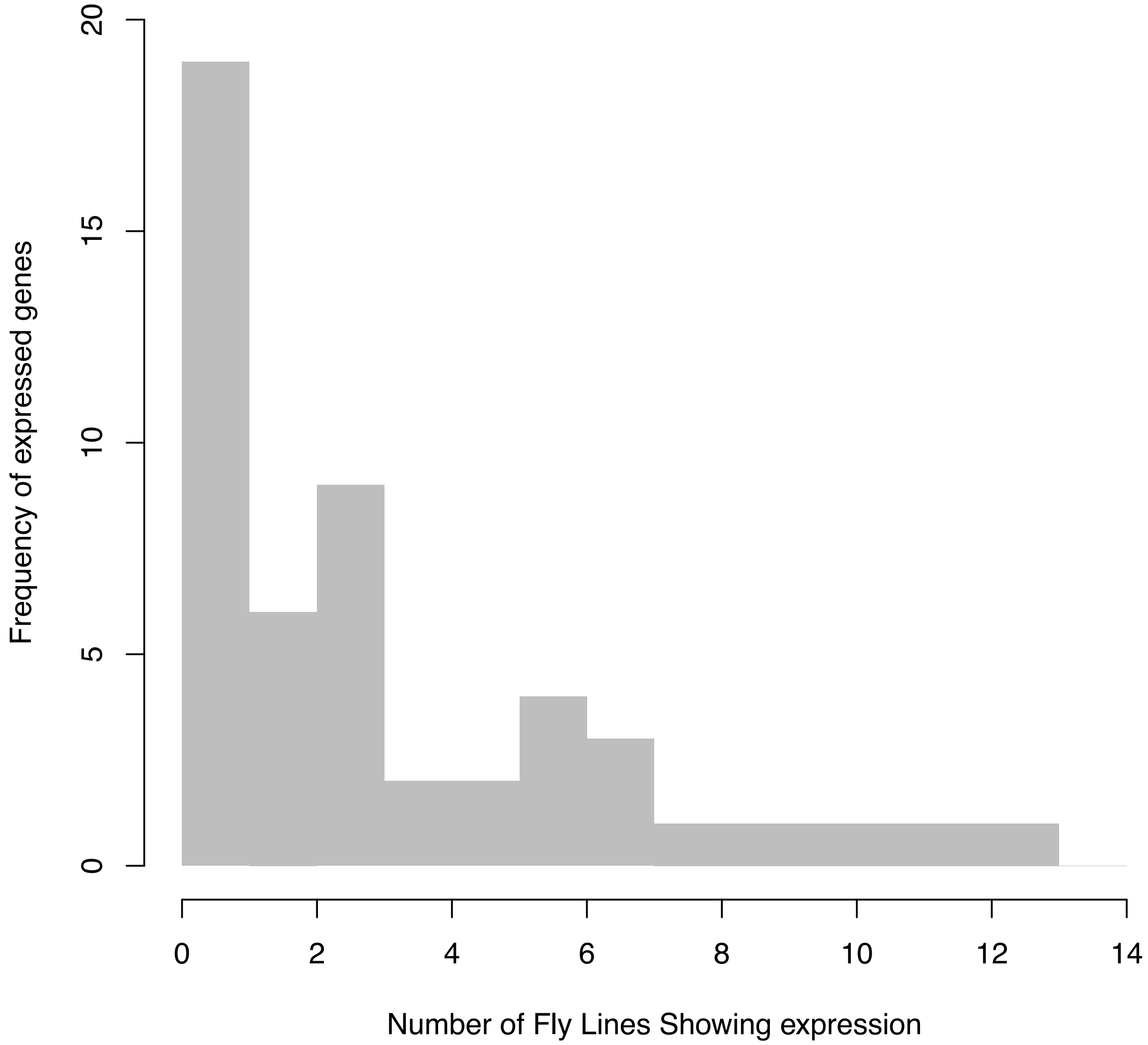

S3 Figure: Site frequency spectrum of rearrangements that are associated with fusion transcripts found in the 14 lines. Most new genes formed at rearrangements are low-frequency variation.
