# Supplementary material for: Chromosomal rearrangements as a source of new gene formation in Drosophila yakuba
Source: PLoS Genet. 2019 Sep 23;15(9):e1008314. doi: 10.1371/journal.pgen.1008314 (PMC6776367; doi:10.1371/journal.pgen.1008314)
Supplement: S4 Fig — (PDF) [file pgen.1008314.s005.pdf]

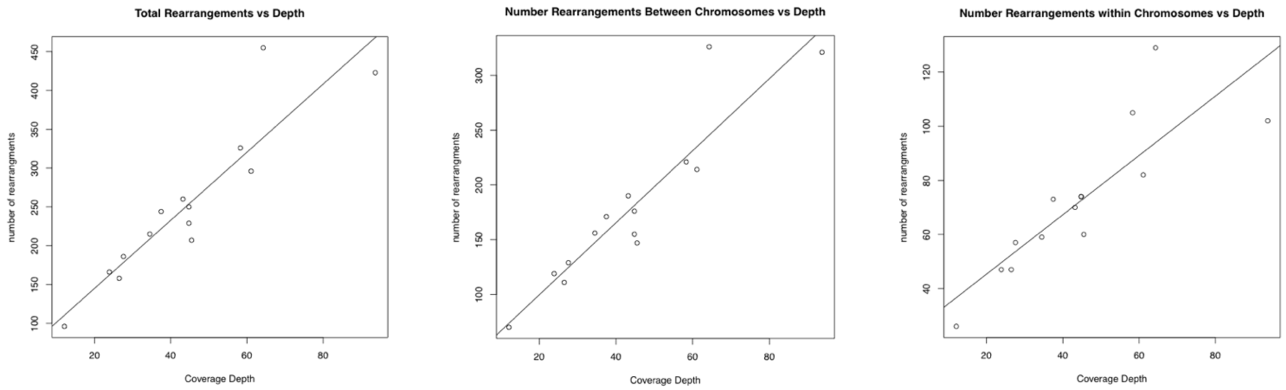

S4 Figure: A) Total, B) between, C) and within chromosome rearrangements identified that have 4 supporting independent read-pairs. There is a strong correlation between sequence coverage depth and total number of rearrangements (R^2=0.8231, P<4.7x10^{-6}), between chromosomes rearrangements (R^2 =0.8443, p<2.2e^{-06}), and within chromosome rearrangements (R^2 =0.6936, P<1.4x10^{-4}).
