# Supplementary material for: Chromosomal rearrangements as a source of new gene formation in Drosophila yakuba
Source: PLoS Genet. 2019 Sep 23;15(9):e1008314. doi: 10.1371/journal.pgen.1008314 (PMC6776367; doi:10.1371/journal.pgen.1008314)
Supplement: S5 Fig — (PDF) [file pgen.1008314.s006.pdf]

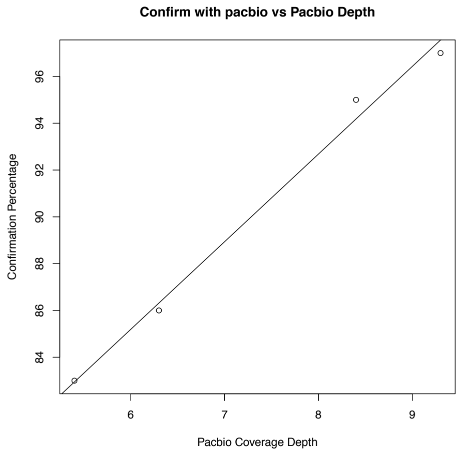

S5 Figure: Percent of the rearrangements identified by illumine paired-end sequencing confirmed by PacBio sequencing over the coverage depth of the PacBio sequencing We aligned PacBio sequence reads to the *D. yakuba* reference using a BLASTn with the repetitive DNA filter turned off and an e-value cutoff of 10^{-10}. If a single molecule read blast within 2kb of the genomic rearrangement call it was counted as confirmation.
