# Supplementary material for: Chromosomal rearrangements as a source of new gene formation in Drosophila yakuba
Source: PLoS Genet. 2019 Sep 23;15(9):e1008314. doi: 10.1371/journal.pgen.1008314 (PMC6776367; doi:10.1371/journal.pgen.1008314)
Supplement: S6 Fig — (PDF) [file pgen.1008314.s007.pdf]

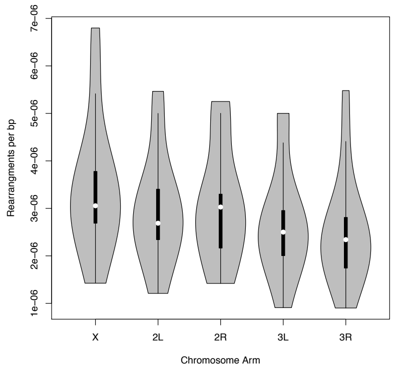

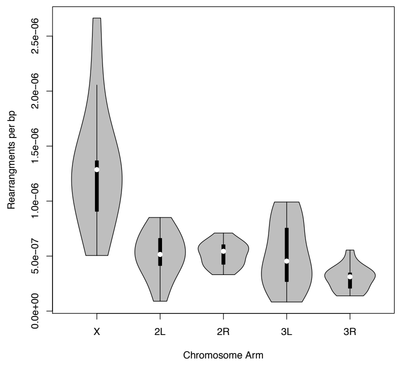

S6 Figure: Number of chromosomal rearrangement breakpoints per base pair on each chromosome arm for each *D. yakuba* line. A) Number of rearrangement brekapoints between chromosomes each rearrangement was counted for both arms involved. Chromosome rearrangements, the X chromosome shows significantly higher number of within chromosome rearrangements with 261 of the 671 total rearrangements (ANOVA, $F(4, 52)= 42.06$, $P<10^{-14}$, Tukey HSD for each X comparison $P<10^{-7}$). While none of the autosomes are significantly different with respect to number of rearrangements per base pair (Tukey HSD for all comparisons, $P>0.05$). B) Number of rearrangement breakpoints with both ends on the same chomosome arm and 1Mb distally between the two ends varied by chromosome arm (ANOVA, $F(4, 52)= 14.26$, $P<10^{-7}$) We found that the X chromosome had significantly more rearrangement sites than chromosome arms 2L (Tukey HSD, $P<0.009$), 3L (Tukey HSD, $P<10^{-5}$), and 3R (Tukey, HSD $P<10^{-6}$) but not 2R (Tukey HSD, $P=0.068$). Chromosome arm 2R showed greater number of between chromosome rearrangements per base pair than chromosome arms 3L (Tukey HSD, $P<0.05$), and 3R (Tukey HSD, $P<0.001$).
