# Supplementary material for: Chromosomal rearrangements as a source of new gene formation in Drosophila yakuba
Source: PLoS Genet. 2019 Sep 23;15(9):e1008314. doi: 10.1371/journal.pgen.1008314 (PMC6776367; doi:10.1371/journal.pgen.1008314)
Supplement: S7 Fig — (PDF) [file pgen.1008314.s008.pdf]

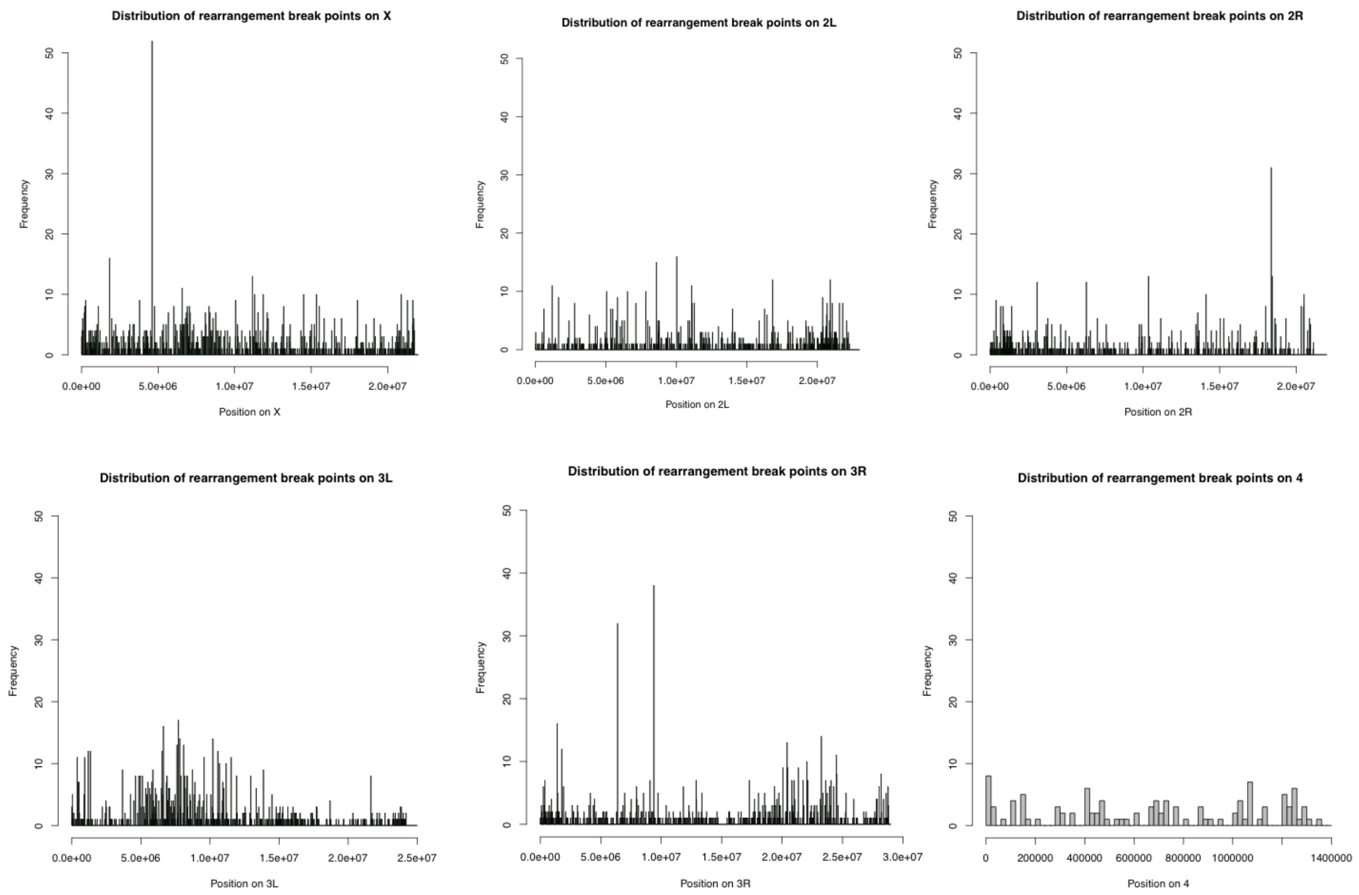

S7 Figure: Distribution of rearrangement sites along the 4 chromosome arms using 20kb windows. There are 4 rearrangement hotspots, one on X, one on 2R, and two on 3R.
