# Supplementary material for: Chromosomal rearrangements as a source of new gene formation in Drosophila yakuba
Source: PLoS Genet. 2019 Sep 23;15(9):e1008314. doi: 10.1371/journal.pgen.1008314 (PMC6776367; doi:10.1371/journal.pgen.1008314)
Supplement: S8 Fig — (PDF) [file pgen.1008314.s009.pdf]

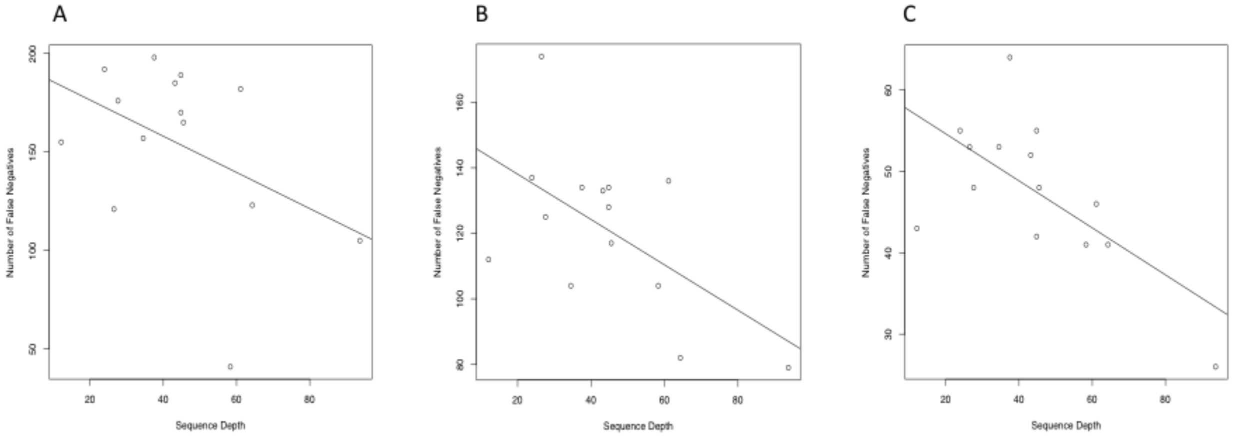

S8 Figure: A) Total Number of possible false negatives of each line against sequence coverage depth of that line. B) Number of possible false negative between chromosomes, C) number of false negatives within chromosome rearrangements. There is not a significant correlation between the total number of false negatives and coverage depth (R^2= 0.1189, *P*=0.1189), however isolating between chromosome and within chromosome rearrangements they both show a negative correlation between coverage depth and false negative estimates (between chromosomes R^2=0.2839, *P*=0.02892; within chromosomes R^2=.3841, *P*=0.0107). Possible false negatives were classified by having 1-3 read pair support for a rearrangement that is found in at least one other line with 4 or greater read pair support.
