# Supplementary material for: Chromosomal rearrangements as a source of new gene formation in Drosophila yakuba
Source: PLoS Genet. 2019 Sep 23;15(9):e1008314. doi: 10.1371/journal.pgen.1008314 (PMC6776367; doi:10.1371/journal.pgen.1008314)
Supplement: S9 Fig — (PDF) [file pgen.1008314.s010.pdf]

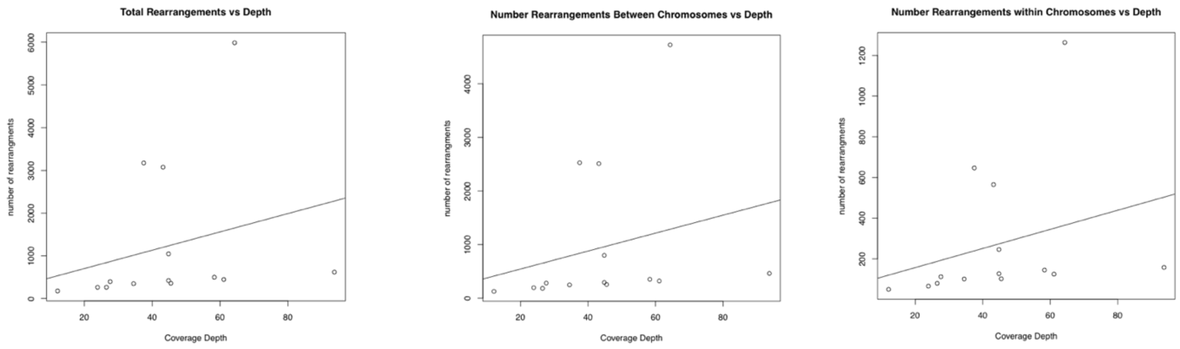

S9 Figure: A) Total, B) between, C) and within chromosome rearrangements identified that have 3 supporting independent read-pairs. There is no correlation between sequence coverage depth and total number of rearrangements (R^2=0.009632, P=0.3678), between chromosomes rearrangements (R^2 =0.0132, P=0.38), and within chromosome rearrangements (R^2 =0.004914, P=0.3226). Lines CY04B, CY08A, CY17C, and CY21B3 have abnormally high amount of structure calls, most are rearrangements that have support of three read-pairs.
