# Supplementary material for: Chromosomal rearrangements as a source of new gene formation in Drosophila yakuba
Source: PLoS Genet. 2019 Sep 23;15(9):e1008314. doi: 10.1371/journal.pgen.1008314 (PMC6776367; doi:10.1371/journal.pgen.1008314)
Supplement: S1 Table — (PDF) [file pgen.1008314.s011.pdf]

S1 Table: New gene formation in strains of *D. yakuba.* Raw totals of chromosomal rearrangements on each chromosome arm that are supported by genomic structure calls and Tophat fusion calls for testes, male carcass, ovaries, and female carcass.

line	X		2L		2R		3L		3R		4		Total
	**within**	**between**	**within**	**between**	**within**	**between**	**within**	**between**	**within**	**between**	**within**	**between**	
NY73 testes	0	1	1	2	2	0	1	0	0	1	0	0	6
NY73 mal car	0	0	1	1	2	0	1	0	0	1	0	0	5
NY73 ovary	0	1	0	0	0	0	1	0	0	1	0	0	2
NY73 fem car	0	0	0	1	0	0	0	0	0	1	0	0	1
NY66 testes	0	3	3	1	0	0	1	5	3	1	0	2	13
NY66 mal car	0	1	1	2	1	0	1	2	0	0	0	3	7
NY66 ovary	0	1	0	0	0	0	1	2	0	1	0	2	4
NY66 fem car	0	0	0	0	0	0	1	0	0	0	0	0	1
NY62 testes	0	5	0	3	3	1	0	2	3	2	0	1	13
NY62 mal car	0	3	0	5	1	0	0	3	0	1	0	0	7
NY62 ovary	0	1	0	0	1	0	0	0	0	1	0	0	2
NY62 fem car	0	0	0	0	0	0	0	0	0	0	0	0	0
NY48 testes	0	0	0	0	1	0	0	0	0	0	0	0	1
NY48 mal car	0	1	0	2	1	0	0	0	0	1	0	0	3
NY48 ovary	0	0	0	0	0	0	0	0	0	0	0	0	0
NY48 fem car	0	0	0	0	0	0	0	0	0	0	0	0	0
NY56 testes	0	1	0	3	2	0	0	2	0	0	0	0	5
NY56 mal car	0	1	0	4	1	0	0	3	0	1	0	1	6
NY56 ovary	0	1	0	0	0	0	0	0	0	1	0	0	1
NY56 fem car	0	1	0	0	0	0	0	1	0	0	0	0	1
NY81 testes	0	2	0	1	3	0	0	0	0	1	0	0	5
NY81 mal car	0	2	0	1	2	0	0	1	0	0	0	0	4
NY81 ovary	0	0	0	0	0	0	0	0	0	0	0	0	0
NY81 fem car	0	0	0	0	0	0	0	0	0	0	0	0	0
NY85 testes	0	1	0	1	2	0	1	0	0	0	0	0	4
NY85 mal car	0	2	3	2	2	0	1	2	2	2	0	0	12
NY85 ovary	0	1	0	0	1	0	0	1	0	2	0	0	3
NY85 fem car	0	0	0	0	0	0	1	1	0	1	0	0	2
CY22B testes	0	2	3	1	2	0	1	2	0	0	0	1	9

CY22B mal car	0	3	0	3	4	0	1	3	0	0	0	1	10
CY22B ovary	0	0	2	0	1	0	0	0	0	0	0	0	3
CY22B fem car	0	1	0	1	0	0	0	0	0	0	0	0	1
CY21B3 testes	0	2	1	0	5	0	1	1	0	1	0	0	9
CY21B3 mal car	0	3	1	1	5	1	1	2	0	0	0	1	11
CY21B3 ovary	0	1	1	0	3	0	0	0	0	1	0	0	5
CY21B3 fem car	0	0	0	0	2	0	0	0	0	0	0	0	2
CY20A testes	0	2	2	1	4	1	0	1	0	3	0	0	10
CY20A mal car	0	1	1	1	2	1	0	2	0	3	0	2	8
CY20A ovary	0	1	0	0	3	1	0	0	0	2	0	0	5
CY20A fem car	0	0	0	0	3	1	0	0	0	1	0	0	4
CY28A4 testes	2	4	2	4	0	0	2	1	0	0	0	1	11
CY28A4 mal car	0	3	1	4	1	0	2	2	0	0	0	3	10
CY28A4 ovary	0	2	0	2	0	0	1	0	0	0	0	0	3
CY28A4 fem car	0	0	0	0	0	0	1	0	0	0	0	0	1
CY04B testes	0	3	2	1	3	0	0	3	1	1	0	2	11
CY04B mal car	0	3	0	1	2	0	0	5	0	0	0	3	8
CY04B ovary	0	1	0	0	2	0	0	0	0	1	0	0	3
CY04B fem car	0	1	0	0	0	0	0	3	0	0	0	2	3
CY17C testes	0	2	1	1	3	0	0	1	0	0	0	0	6
CY17C mal car	0	2	1	6	3	0	0	2	1	4	0	0	12
CY17C ovary	0	0	0	0	0	0	0	0	0	0	0	0	0
CY17C fem car	0	0	0	0	0	0	0	1	0	0	0	1	1
CY08A testes	0	2	0	2	3	0	0	0	1	0	0	0	6
CY08A mal car	0	3	1	3	3	0	0	2	0	1	0	1	9
CY08A ovary	0	1	1	0	1	0	0	0	0	1	0	0	3
CY08A fem car	0	0	1	0	0	0	0	0	0	0	0	0	1
