# Supplementary material for: Chromosomal rearrangements as a source of new gene formation in Drosophila yakuba
Source: PLoS Genet. 2019 Sep 23;15(9):e1008314. doi: 10.1371/journal.pgen.1008314 (PMC6776367; doi:10.1371/journal.pgen.1008314)
Supplement: S2 Table — (PDF) [file pgen.1008314.s012.pdf]

S2 Table: Number of genes within 1kb of rearrangement calls that are either down regulated or up regulated in each individual tissue.

Tissue	Up regulated	Down Regulated	Both*
**Testes**	18	21	2
**Male Carcass**	15	30	6
**Ovaries**	16	32	2
**Female Carcass**	18	18	0

* Both indicates that in some lines that have a certain rearrangement near the gene has up regulated expression while in other lines that have the rearrangement the gene is down regulated.
