# Supplementary material for: Chromosomal rearrangements as a source of new gene formation in Drosophila yakuba
Source: PLoS Genet. 2019 Sep 23;15(9):e1008314. doi: 10.1371/journal.pgen.1008314 (PMC6776367; doi:10.1371/journal.pgen.1008314)
Supplement: S3 Table — (PDF) [file pgen.1008314.s013.pdf]

S3 Table: Tukey multiple comparisons of means of total rearrangement sites found per base pair on each major chromosome arm

	Differential	Lower end point	Upper end point	p adj
2R-2L	1.15E-07	-5.55E-07	7.86E-07	0.9883147
3L-2L	-2.85E-07	-9.56E-07	3.86E-07	0.7509075
3R-2L	-8.73E-07	-1.54E-06	-2.02E-07	0.0048835
X-2L	2.06E-06	1.39E-06	2.73E-06	0
3L-2R	-4.00E-07	-1.07E-06	2.70E-07	0.4507802
3R-2R	-9.88E-07	-1.66E-06	-3.17E-07	0.0010817
X-2R	1.94E-06	1.27E-06	2.62E-06	0
3R-3L	-5.88E-07	-1.26E-06	8.31E-08	0.1120626
X-3L	2.35E-06	1.67E-06	3.02E-06	0
X-3R	2.93E-06	2.26E-06	3.60E-06	0
