# Supplementary material for: Chromosomal rearrangements as a source of new gene formation in Drosophila yakuba
Source: PLoS Genet. 2019 Sep 23;15(9):e1008314. doi: 10.1371/journal.pgen.1008314 (PMC6776367; doi:10.1371/journal.pgen.1008314)
Supplement: S4 Table — (PDF) [file pgen.1008314.s014.pdf]

S4 Table: Pairwise comparisons between chromosome arms in abundance of rearrangements within chromosomes

	Differential	Lower end point	Upper end point	p adj
2R-2L	5.06E-09	-2.37E-07	2.47E-07	0.9999971
3L-2L	-7.15E-09	-2.49E-07	2.35E-07	0.9999886
3R-2L	-2.12E-07	-4.54E-07	2.99E-08	0.1116779
X-2L	8.00E-07	5.58E-07	1.04E-06	0
3L-2R	-1.22E-08	-2.54E-07	2.30E-07	0.9999037
3R-2R	-2.17E-07	-4.59E-07	2.48E-08	0.0981546
X-2R	7.95E-07	5.53E-07	1.04E-06	0
3R-3L	-2.05E-07	-4.47E-07	3.70E-08	0.1332882
X-3L	8.08E-07	5.66E-07	1.05E-06	0
X-3R	1.01E-06	7.71E-07	1.25E-06	0
