# Supplementary material for: Chromosomal rearrangements as a source of new gene formation in Drosophila yakuba
Source: PLoS Genet. 2019 Sep 23;15(9):e1008314. doi: 10.1371/journal.pgen.1008314 (PMC6776367; doi:10.1371/journal.pgen.1008314)
Supplement: S5 Table — (PDF) [file pgen.1008314.s015.pdf]

S5 Table: pairwise comparisons between chromosome arms in abundance of rearrangements between chromosomes

	Differential	Lower end point	Upper end point	p adj
2R-2L	1.05E-07	-2.66E-07	4.76E-07	0.9287468
3L-2L	-2.71E-07	-6.42E-07	1.00E-07	0.2520333
3R-2L	-4.48E-07	-8.19E-07	-7.73E-08	0.0104721
X-2L	4.59E-07	8.83E-08	8.30E-07	0.008239
3L-2R	-3.76E-07	-7.47E-07	-5.06E-09	0.0454781
3R-2R	-5.54E-07	-9.24E-07	-1.83E-07	0.0009076
X-2R	3.54E-07	-1.70E-08	7.25E-07	0.0682095
3R-3L	-1.78E-07	-5.48E-07	1.93E-07	0.659954
X-3L	7.30E-07	3.59E-07	1.10E-06	0.0000091
X-3R	9.07E-07	5.37E-07	1.28E-06	0.0000001
