# Supplementary material for: Chromosomal rearrangements as a source of new gene formation in Drosophila yakuba
Source: PLoS Genet. 2019 Sep 23;15(9):e1008314. doi: 10.1371/journal.pgen.1008314 (PMC6776367; doi:10.1371/journal.pgen.1008314)
Supplement: S6 Table — (PDF) [file pgen.1008314.s016.pdf]

S6 Table: Total number of rearrangement sites that have coverage depth twice the average coverage depth of each line. Coverage depth of each rearrangement plus 100 base pair regions or 500 base pair regions flanking each rearrangement site were calculated and compared to the average coverage depth. The number of sites that had double the coverage depth divided by the total number of rearrangement sites for that line is represented in the percent columns.

	**Average Coverage depth**	**Total sites**	**500 bp**	**500 bp percent**	**100 bp**	**100 bp percent**
NY48	34.5	430	33	7.6	91	21.2
*NY56**	12.1	192	69	35.9	131	68.2
NY62	44.8	502	33	6.6	80	15.9
NY66	26.5	316	33	8.9	71	22.5
NY73	27.6	372	39	10.5	91	24.5
NY81	23.9	332	24	7.2	61	18.4
NY85	61.1	592	19	3.2	48	8.1
CY08A	37.5	520	21	4	37	7.1
CY20A	93.7	846	21	2.5	88	10.4
CY28A4	58.3	652	25	3.8	85	13
CY04B	64.3	910	25	2.7	84	9.2
CY22B	45.5	414	8	1.9	33	8
CY21B3	44.8	458	6	1.3	36	7.9
CY17C	43.2	488	13	2.7	34	7

*with average depth of only 12.1, the total number of rearrangements are more likely an over representation than the total number of possible duplications.
