# Supplementary material for: Chromosomal rearrangements as a source of new gene formation in Drosophila yakuba
Source: PLoS Genet. 2019 Sep 23;15(9):e1008314. doi: 10.1371/journal.pgen.1008314 (PMC6776367; doi:10.1371/journal.pgen.1008314)
Supplement: S7 Table — (PDF) [file pgen.1008314.s017.pdf]

S7 Table: Number and ratios in which the abnormal read pairs were aligned in the same direction. The reads lining in the same direction indicates either the translocation was inserted inverted or (if within chromosome) the rearrangement identified is an inversion.

Line	Coverage depth	Total Within chromosomes	Within chromosomes linear reads	Within chromosomes Percent	Total Between chromosomes	Between chromosomes linear reads	Between chromosome Percent
NY73	27.6	57	31	54%	129	62	48%
NY66	26.5	47	22	47%	111	60	54%
NY62	44.8	74	39	53%	177	96	54%
NY48	34.5	59	35	59%	156	66	42%
NY56	12.1	26	14	54%	70	33	47%
NY81	23.9	47	28	60%	119	55	46%
NY85	61.1	82	46	56%	214	97	45%
CY22B	45.5	60	24	40%	148	62	42%
CY21B3	44.8	74	35	47%	155	76	49%
CY20A	93.7	102	48	47%	321	155	48%
CY28A4	58.3	105	52	50%	221	114	52%
CY04B	64.3	129	73	57%	326	150	46%
CY17C	43.2	73	33	45%	171	82	48%
CY08A	37.5	70	36	51%	190	81	43%
