# Supplementary material for: Chromosomal rearrangements as a source of new gene formation in Drosophila yakuba
Source: PLoS Genet. 2019 Sep 23;15(9):e1008314. doi: 10.1371/journal.pgen.1008314 (PMC6776367; doi:10.1371/journal.pgen.1008314)
Supplement: S8 Table — (PDF) [file pgen.1008314.s018.pdf]

**S8 Table:** GO terms all genes with *D. melanogaster* ortholog (733) associated with rearrangements

| Cluster | count | enrichment | P_value | Benjamini |
|---|---|---|---|---|
| Alternative Splicing | 82 | 8.39 | 4.30E-14 | 1.00E-11 |
| Membrane | 240 | 7.18 | 1.60E-09 | 1.90E-07 |
| Glycoprotein | 53 | 3.56 | 2.20E-06 | 7.70E-05 |
| Pleckstrin | 22 | 3.21 | 6.00E-05 | 2.10E-02 |
| SH3 homology/domain | 14 | 2.7 | 1.80E-04 | 3.20E-02 |
| protein kinase/transferase | 38 | 2.4 | 1.00E-07 | 3.60E-05 |
| dorsal closure head/eye development | 16 | 2.93 | 4.00E-04 | 3.80E-02 |
| PDZ domain | 13 | 2. 06 | 1.80E-04 | 1.80E-012 |
