# Supplementary material for: Chromosomal rearrangements as a source of new gene formation in Drosophila yakuba
Source: PLoS Genet. 2019 Sep 23;15(9):e1008314. doi: 10.1371/journal.pgen.1008314 (PMC6776367; doi:10.1371/journal.pgen.1008314)
Supplement: S1 Data — (ZIP) [file pgen.1008314.s019.zip › Denovogenes/SupplementalRNASeqFig.pdf]

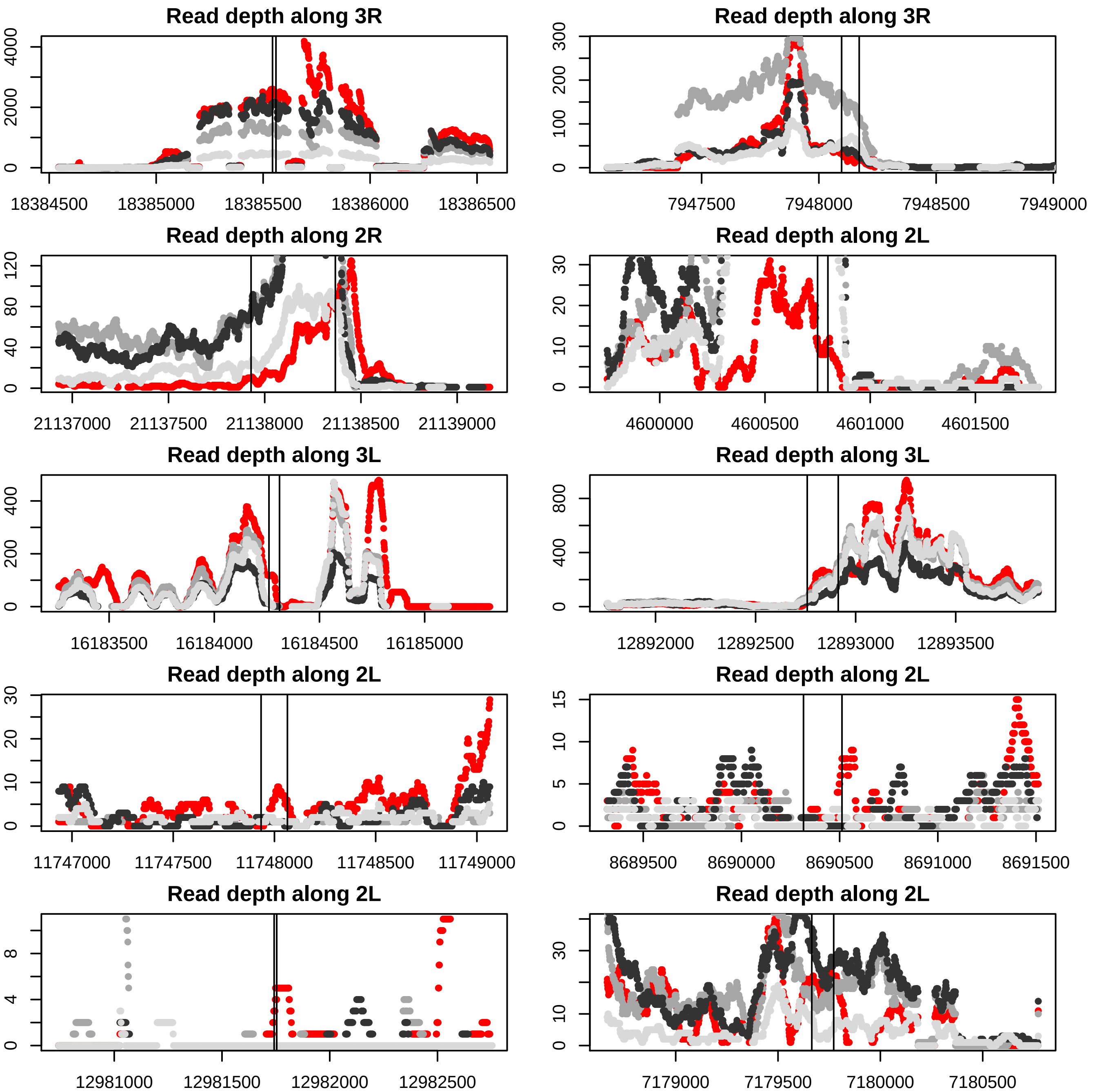
Read depth along 3R
Read depth along 3R
Read depth along 2R
Read depth along 2L
Read depth along 3L
Read depth along 3L
Read depth along 2L
Read depth along 2L
Read depth along 2L
Read depth along 2L
18384500
18385000
18385500
18386000
18386500
7947500
7948000
7948500
7949000
21137000
21137500
21138000
21138500
21139000
4600000
4600500
4601000
4601500
16183500
16184000
16184500
16185000
12892000
12892500
12893000
12893500
11747000
11747500
11748000
11748500
11749000
8689500
8690000
8690500
8691000
8691500
12981000
12981500
12982000
12982500
7179000
7179500
7180000
7180500

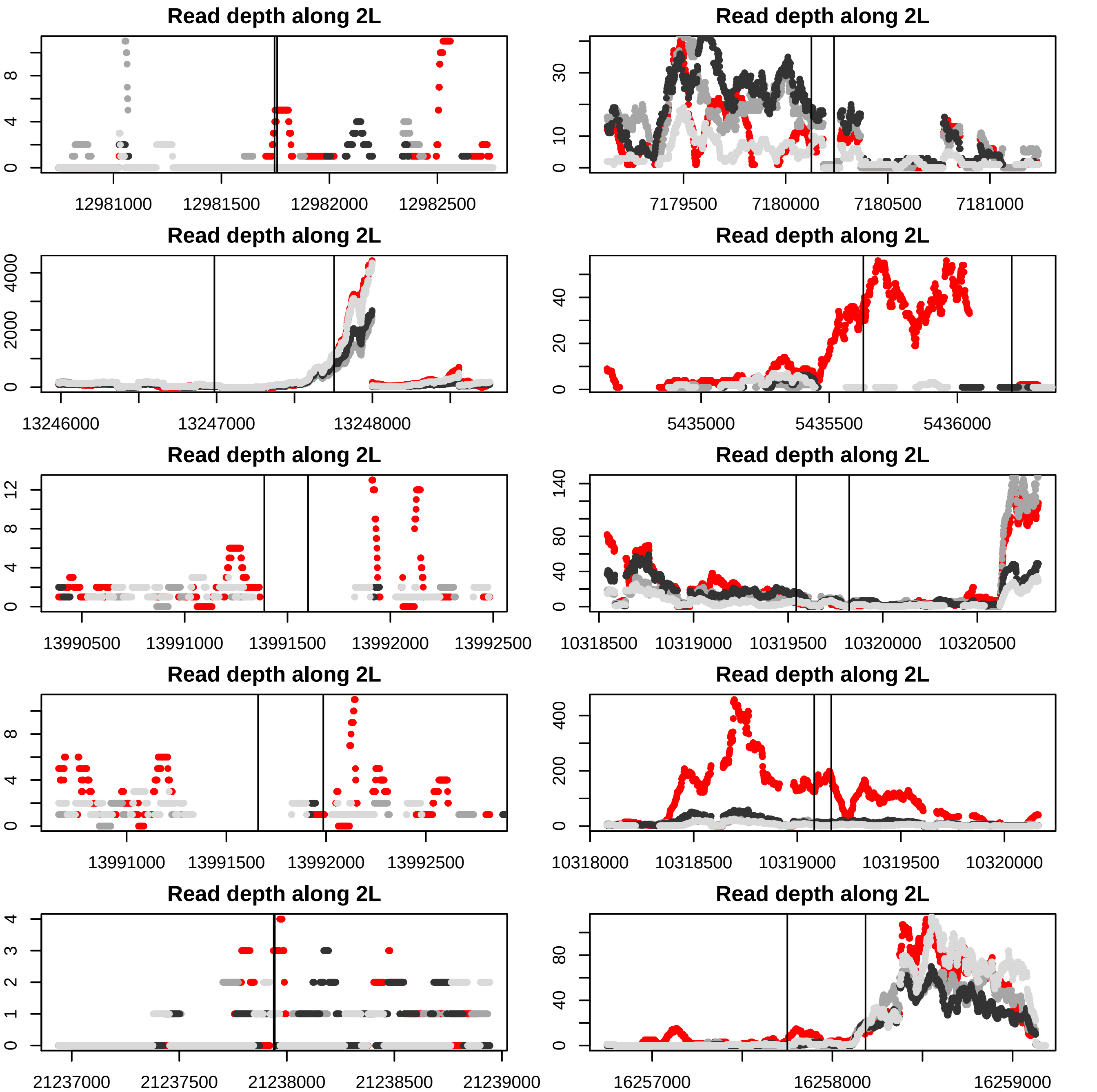
Read depth along 2L
0
4
8
12981000
12981500
12982000
12982500
Read depth along 2L
0
10
30
7179500
7180000
7180500
7181000
Read depth along 2L
0
2000
4000
13246000
13247000
13248000
Read depth along 2L
0
20
40
5435000
5435500
5436000
Read depth along 2L
0
4
8
12
13990500
13991000
13991500
13992000
13992500
Read depth along 2L
0
40
80
140
10318500
10319000
10319500
10320000
10320500
Read depth along 2L
0
4
8
13991000
13991500
13992000
13992500
Read depth along 2L
0
200
400
10318000
10318500
10319000
10319500
10320000
Read depth along 2L
0
1
2
3
4
21237000
21237500
21238000
21238500
21239000
Read depth along 2L
0
40
80
16257000
16258000
16259000

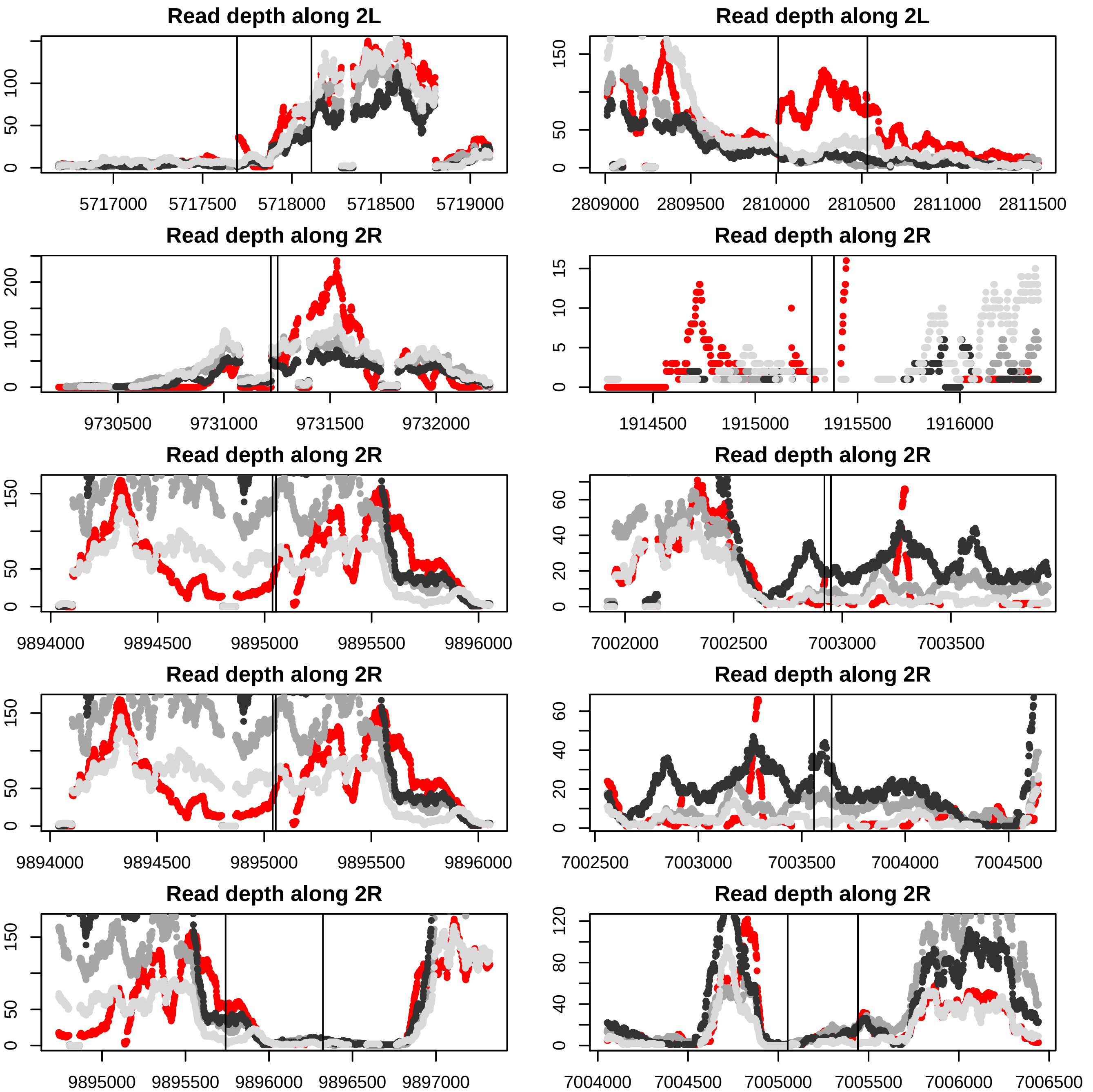
Read depth along 2L
0
50
100
5717000
5717500
5718000
5718500
5719000
Read depth along 2L
0
50
150
2809000
2809500
2810000
2810500
2811000
2811500
Read depth along 2R
0
100
200
9730500
9731000
9731500
9732000
Read depth along 2R
0
5
10
15
1914500
1915000
1915500
1916000
Read depth along 2R
0
50
150
9894000
9894500
9895000
9895500
9896000
Read depth along 2R
0
20
40
60
7002000
7002500
7003000
7003500
Read depth along 2R
0
50
150
9894000
9894500
9895000
9895500
9896000
Read depth along 2R
0
20
40
60
7002500
7003000
7003500
7004000
7004500
Read depth along 2R
0
50
150
9895000
9895500
9896000
9896500
9897000
Read depth along 2R
0
40
80
120
7004000
7004500
7005000
7005500
7006000
7006500

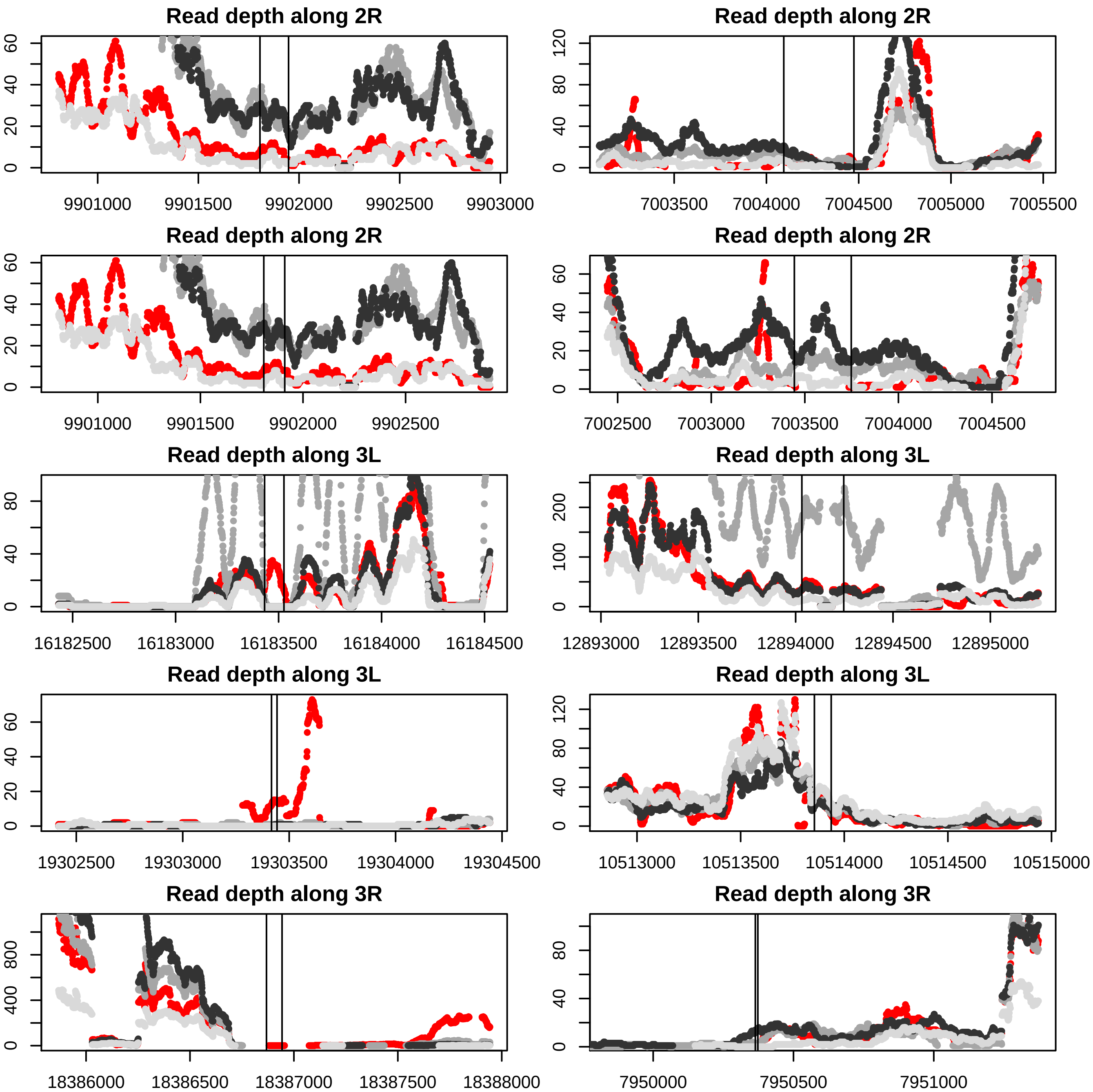
Read depth along 2R
0 20 40 60
9901000 9901500 9902000 9902500 9903000
Read depth along 2R
0 40 80 120
7003500 7004000 7004500 7005000 7005500
Read depth along 2R
0 20 40 60
9901000 9901500 9902000 9902500
Read depth along 2R
0 20 40 60
7002500 7003000 7003500 7004000 7004500
Read depth along 3L
0 40 80
16182500 16183000 16183500 16184000 16184500
Read depth along 3L
0 100 200
12893000 12893500 12894000 12894500 12895000
Read depth along 3L
0 20 40 60
19302500 19303000 19303500 19304000 19304500
Read depth along 3L
0 40 80 120
10513000 10513500 10514000 10514500 10515000
Read depth along 3R
0 400 800
18386000 18386500 18387000 18387500 18388000
Read depth along 3R
0 40 80
7950000 7950500 7951000

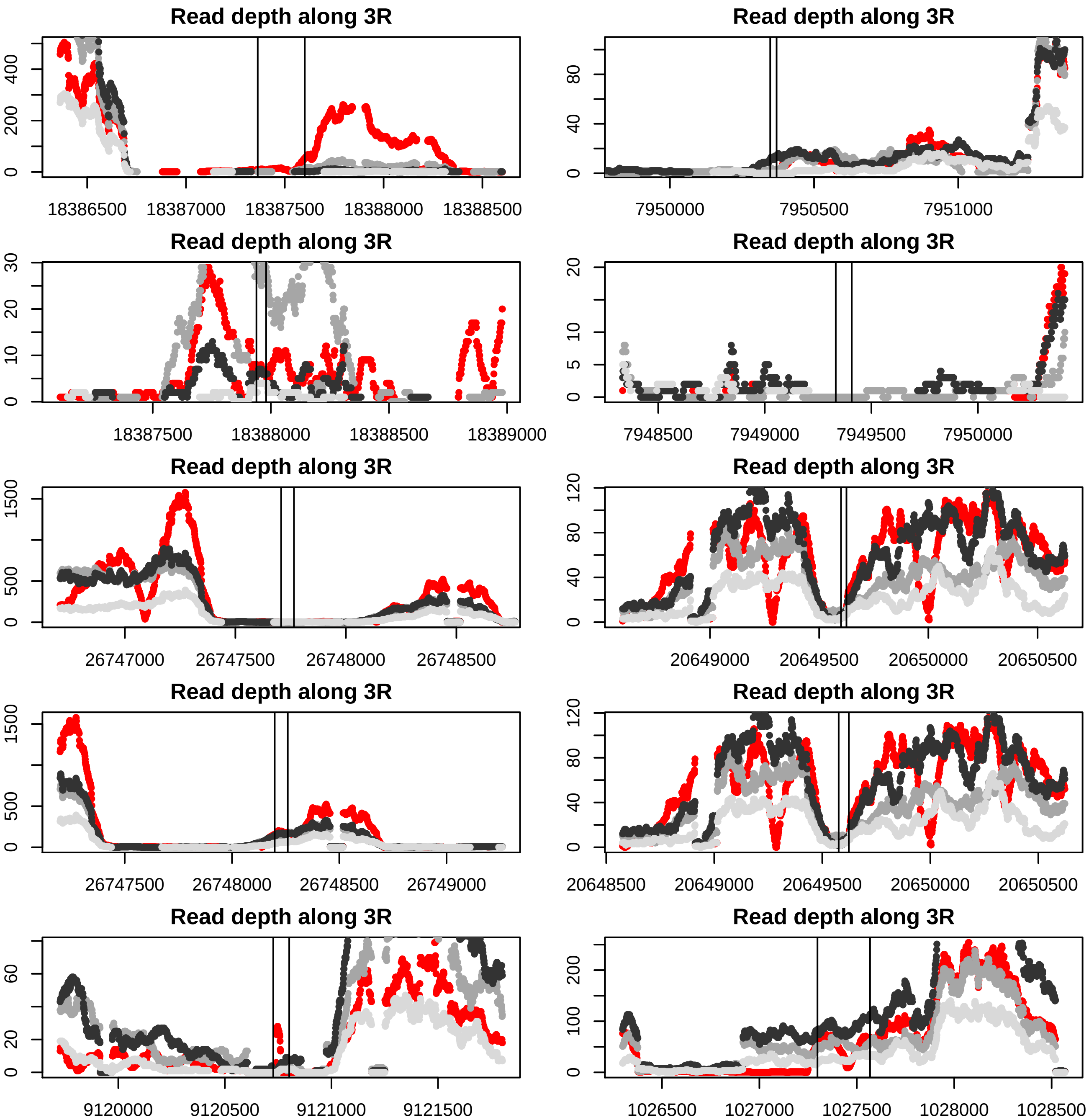
Read depth along 3R
0 200 400
18386500 18387000 18387500 18388000 18388500
Read depth along 3R
0 40 80
7950000 7950500 7951000
Read depth along 3R
0 10 20 30
18387500 18388000 18388500 18389000
Read depth along 3R
0 5 10 20
7948500 7949000 7949500 7950000
Read depth along 3R
0 500 1500
26747000 26747500 26748000 26748500
Read depth along 3R
0 40 80 120
20649000 20649500 20650000 20650500
Read depth along 3R
0 500 1500
26747500 26748000 26748500 26749000
Read depth along 3R
0 40 80 120
20648500 20649000 20649500 20650000 20650500
Read depth along 3R
0 20 60
9120000 9120500 9121000 9121500
Read depth along 3R
0 100 200
1026500 1027000 1027500 1028000 1028500

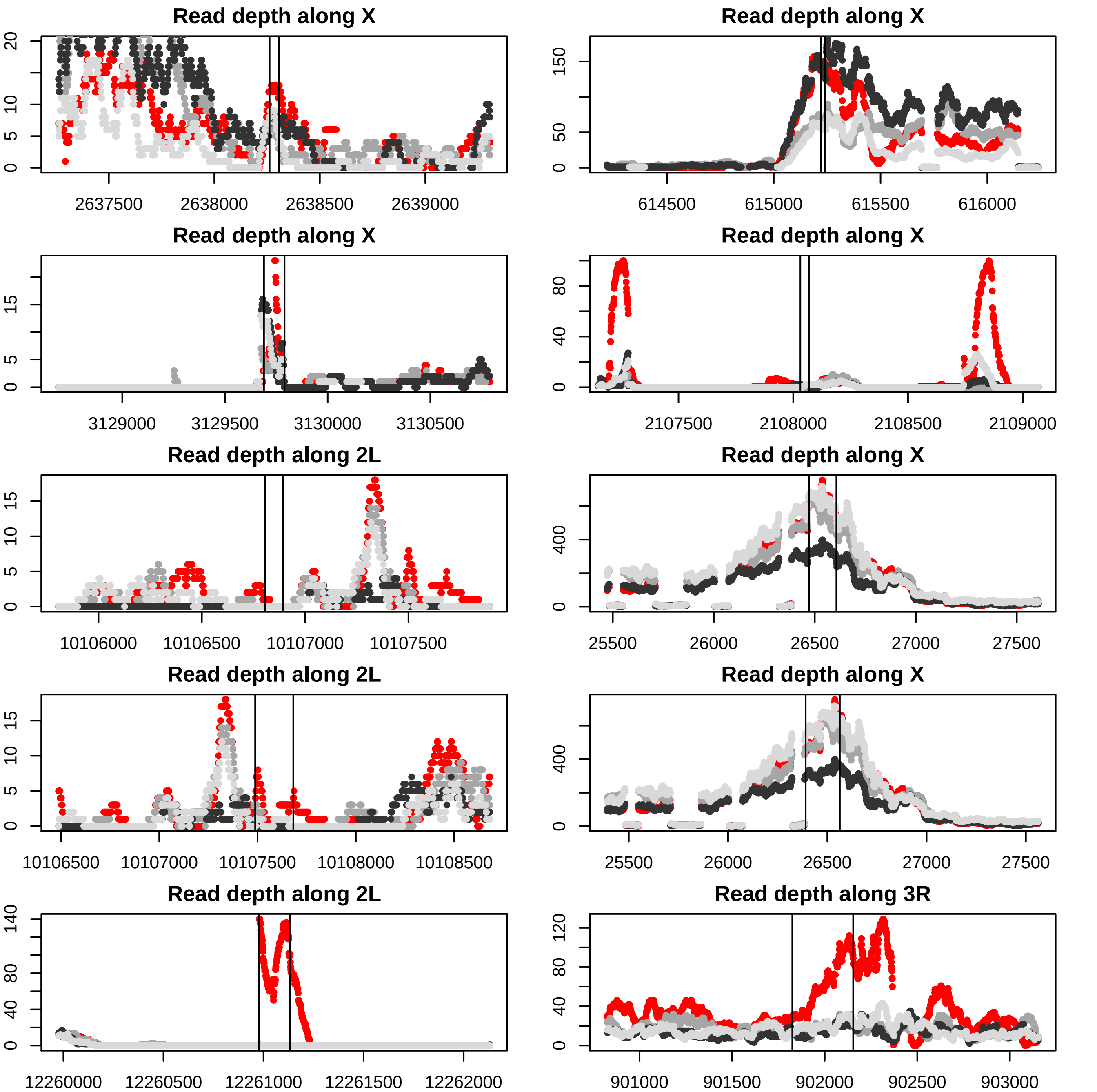
Read depth along X
20
10
5
0
2637500
2638000
2638500
2639000
Read depth along X
150
50
0
614500
615000
615500
616000
Read depth along X
15
5
0
3129000
3129500
3130000
3130500
Read depth along X
80
40
0
2107500
2108000
2108500
2109000
Read depth along 2L
15
10
5
0
10106000
10106500
10107000
10107500
Read depth along X
400
0
25500
26000
26500
27000
27500
Read depth along 2L
15
10
5
0
10106500
10107000
10107500
10108000
10108500
Read depth along X
400
0
25500
26000
26500
27000
27500
Read depth along 2L
140
80
40
0
12260000
12260500
12261000
12261500
12262000
Read depth along 3R
120
80
40
0
901000
901500
902000
902500
903000

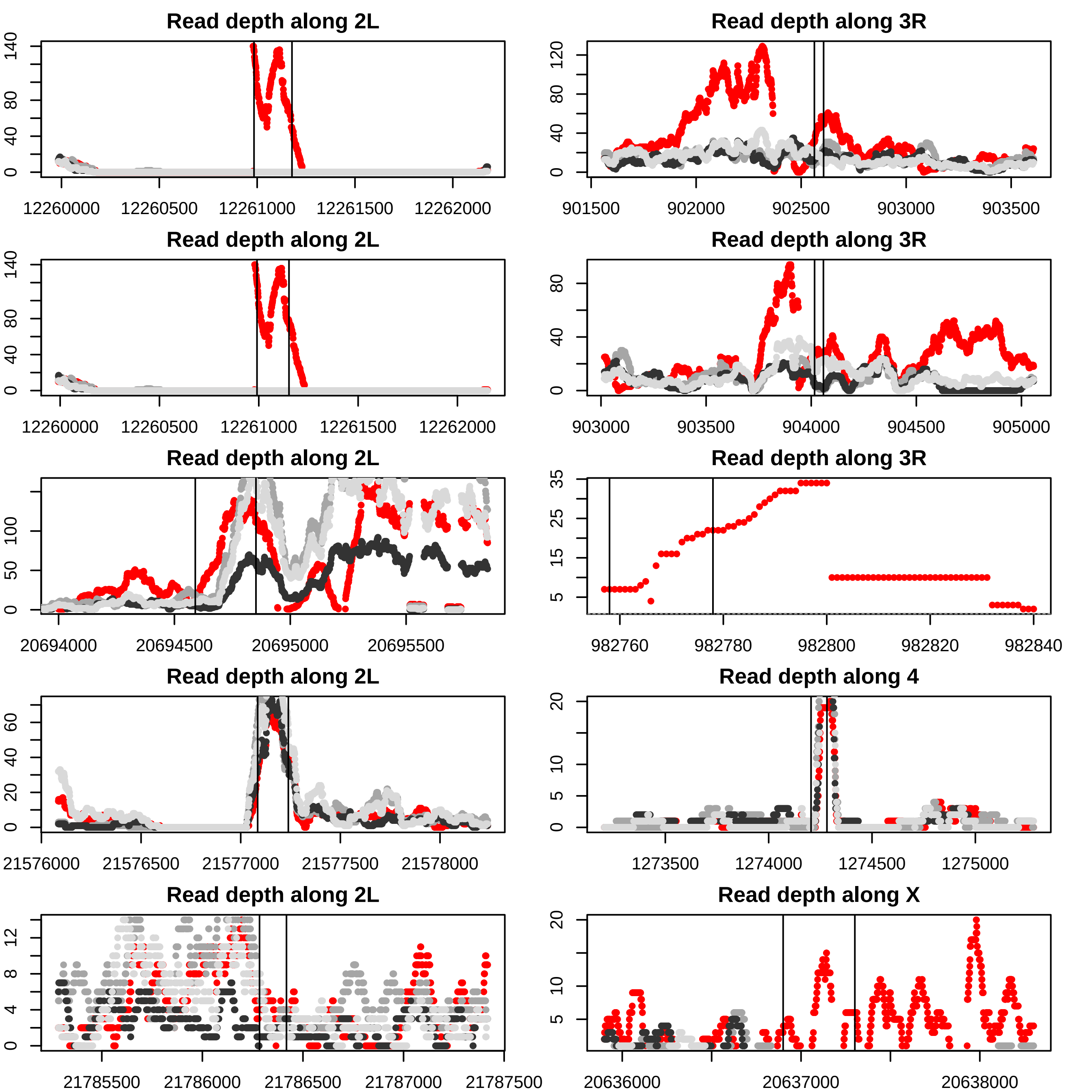
Read depth along 2L
0
40
80
140
12260000
12260500
12261000
12261500
12262000
Read depth along 3R
0
40
80
120
901500
902000
902500
903000
903500
Read depth along 2L
0
40
80
140
12260000
12260500
12261000
12261500
12262000
Read depth along 3R
0
40
80
903000
903500
904000
904500
905000
Read depth along 2L
0
50
100
20694000
20694500
20695000
20695500
Read depth along 3R
5
15
25
35
982760
982780
982800
982820
982840
Read depth along 2L
0
20
40
60
21576000
21576500
21577000
21577500
21578000
Read depth along 4
0
5
10
20
1273500
1274000
1274500
1275000
Read depth along 2L
0
4
8
12
21785500
21786000
21786500
21787000
21787500
Read depth along X
5
10
20
20636000
20637000
20638000

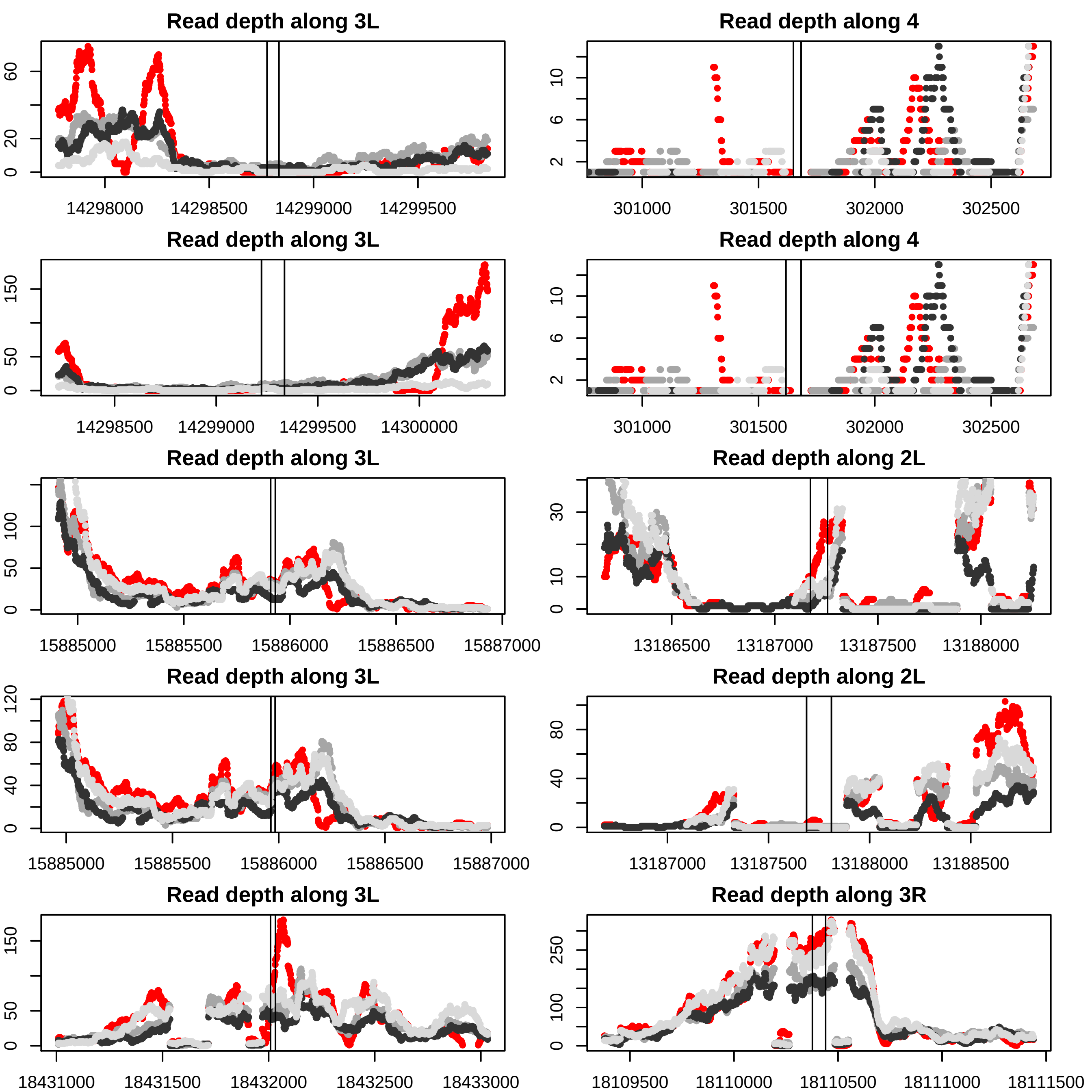
Read depth along 3L
0
20
60
14298000
14298500
14299000
14299500
Read depth along 4
2
6
10
301000
301500
302000
302500
Read depth along 3L
0
50
150
14298500
14299000
14299500
14300000
Read depth along 4
2
6
10
301000
301500
302000
302500
Read depth along 3L
0
50
100
15885000
15885500
15886000
15886500
15887000
Read depth along 2L
0
10
30
13186500
13187000
13187500
13188000
Read depth along 3L
0
40
80
120
15885000
15885500
15886000
15886500
15887000
Read depth along 2L
0
40
80
13187000
13187500
13188000
13188500
Read depth along 3L
0
50
150
18431000
18431500
18432000
18432500
18433000
Read depth along 3R
0
100
250
18109500
18110000
18110500
18111000
18111500

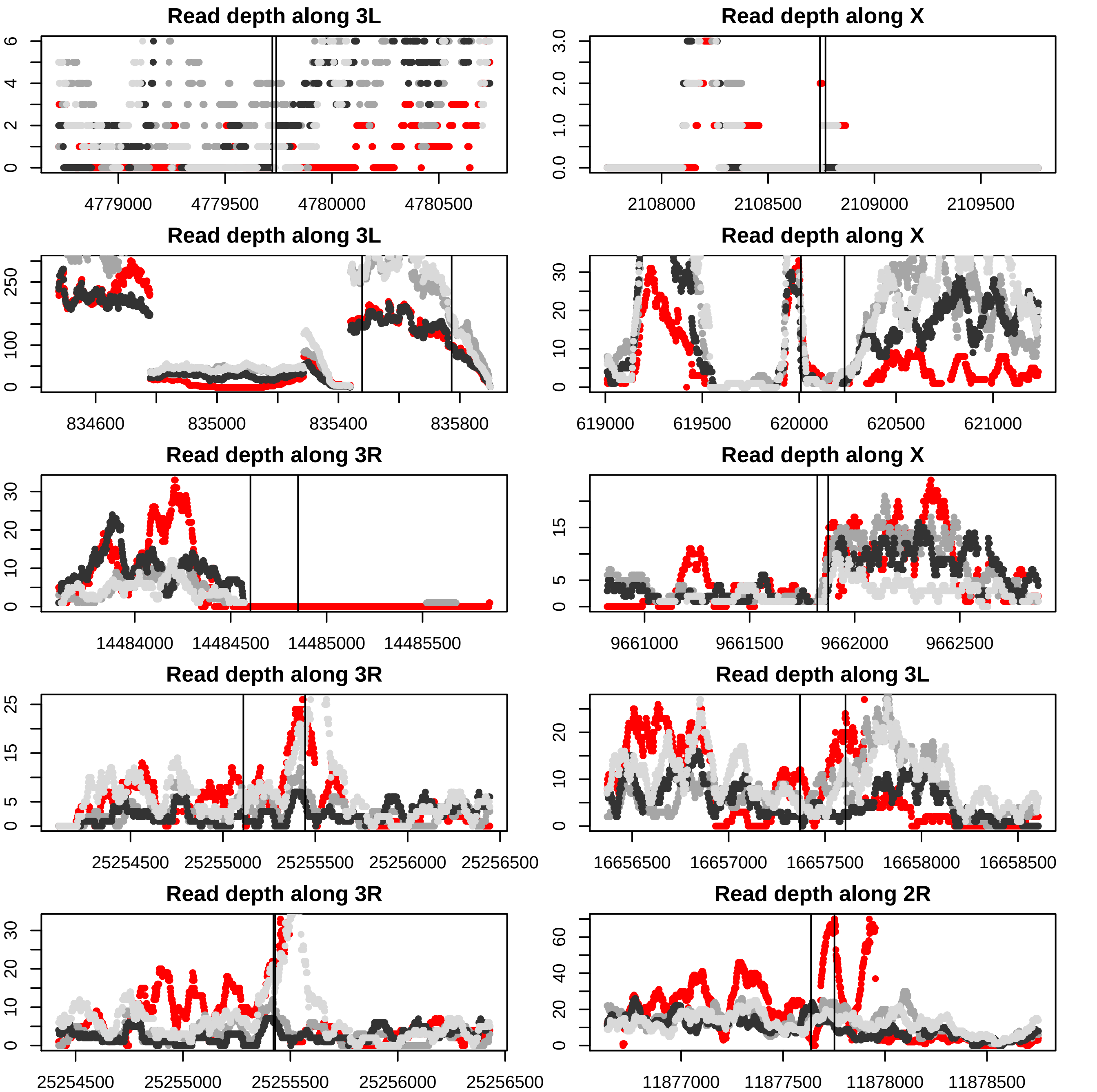
Read depth along 3L
0
2
4
6
4779000
4779500
4780000
4780500
Read depth along X
0.0
1.0
2.0
3.0
2108000
2108500
2109000
2109500
Read depth along 3L
0
100
250
834600
835000
835400
835800
Read depth along X
0
10
20
30
619000
619500
620000
620500
621000
Read depth along 3R
0
10
20
30
14484000
14484500
14485000
14485500
Read depth along X
0
5
15
9661000
9661500
9662000
9662500
Read depth along 3R
0
5
15
25
25254500
25255000
25255500
25256000
25256500
Read depth along 3L
0
10
20
16656500
16657000
16657500
16658000
16658500
Read depth along 3R
0
10
20
30
25254500
25255000
25255500
25256000
25256500
Read depth along 2R
0
20
40
60
11877000
11877500
11878000
11878500

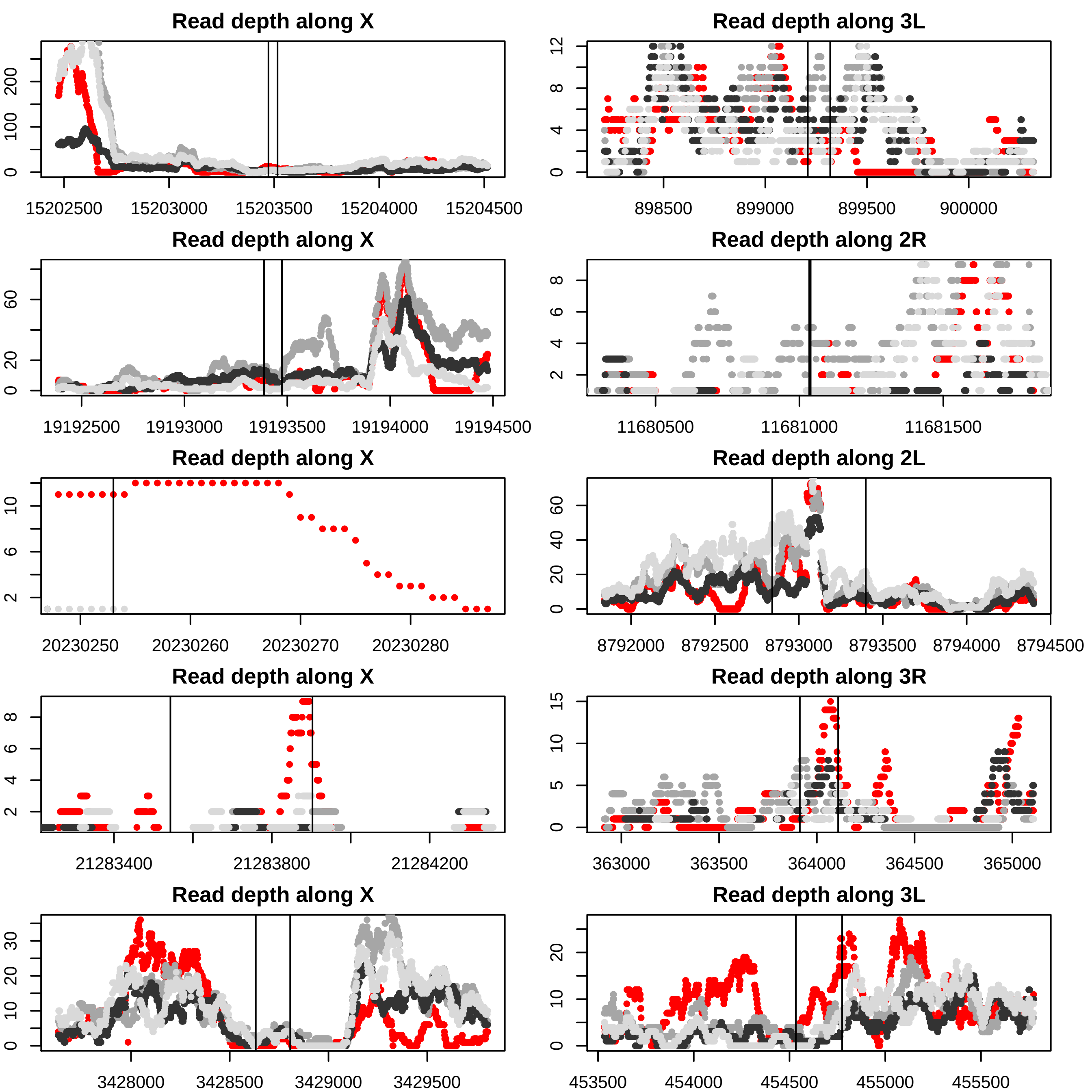
Read depth along X
Read depth along 3L
Read depth along X
Read depth along 2R
Read depth along X
Read depth along 2L
Read depth along X
Read depth along 3R
Read depth along X
Read depth along 3L
15202500
15203000
15203500
15204000
15204500
898500
899000
899500
900000
19192500
19193000
19193500
19194000
19194500
11680500
11681000
11681500
20230250
20230260
20230270
20230280
8792000
8792500
8793000
8793500
8794000
8794500
21283400
21283800
21284200
363000
363500
364000
364500
365000
3428000
3428500
3429000
3429500
453500
454000
454500
455000
455500

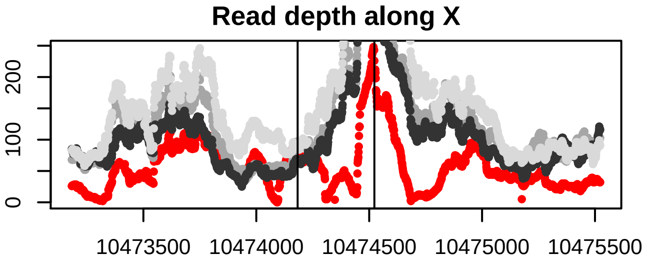
Read depth along X
0
100
200
10473500
10474000
10474500
10475000
10475500

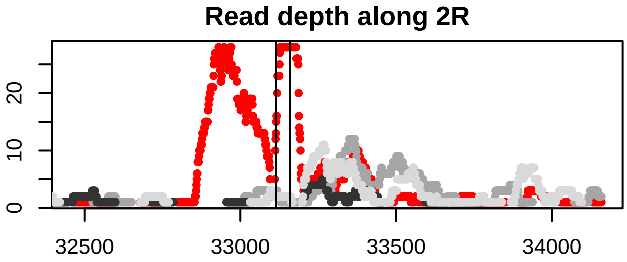
Read depth along 2R
0
10
20
32500
33000
33500
34000
